# Artificial intelligence for predicting the pubertal growth spurt using cephalometric and hand–wrist radiographs: a systematic review and meta-analysis

**DOI:** 10.1186/s12903-026-08627-6

**Published:** 2026-06-03

**Authors:** Jordana Chaves, Matheus Ruffini, Adolfo Souza, Clara Moriguchi, Davi Henrique Ribeiro, Diego Moura, Gabriela Maranhão, Gabrielle Gomes, Maria Eduarda Leal, Juliana Duarte

**Affiliations:** Hospital de Clínicas de Porto Alegre (HCPA), Porto Alegre, RS 90035-903 Brazil

**Keywords:** Artificial intelligence, Machine learning, Cephalometry, Hand–wrist radiograph, Pubertal growth spurt, Skeletal maturation, Growth prediction, Orthodontic timing

## Abstract

**Introduction:**

The optimal timing of dentofacial orthopedic and growth-modifying orthodontic interventions depends on accurate assessment of skeletal maturation, particularly the pubertal growth spurt (PGS). This systematic review and meta-analysis evaluated the performance of artificial intelligence (AI) models for identifying the PGS from cephalometric and hand–wrist radiographs and compared their diagnostic performance across imaging modalities and algorithm types.

**Methods:**

A PRISMA 2020–guided systematic review and meta-analysis was conducted and prospectively registered in PROSPERO (CRD42024594040). PubMed, Embase, Web of Science, and LILACS were searched through December 2025. Eligible studies included individuals aged ≤ 21 years in whom AI was applied to cephalometric and/or hand–wrist radiographs to predict the PGS or classify validated skeletal maturation stages associated with it. Two reviewers independently screened studies, extracted data, and assessed methodological quality using QUADAS-AI. Confusion matrices were extracted or reconstructed, skeletal maturation stages were harmonized into a three-class scheme (pre-spurt, spurt, and post-spurt), and the best-performing model from each study was synthesized using random-effects, bivariate, and hierarchical summary receiver operating characteristic (HSROC) models.

**Results:**

Twenty-one studies published between 2020 and 2025 were included in the quantitative synthesis. The pooled accuracy was 0.83 (95% confidence interval, 0.76–0.89), with substantial between-study heterogeneity. HSROC analysis including 20 studies showed good overall discriminative ability, with summary sensitivity and specificity estimates of 0.76 and 0.88, respectively; complementary bivariate pooling yielded estimates of 0.86 and 0.93, respectively. In subgroup analyses, hand–wrist-based models showed higher sensitivity, whereas cephalometric models showed higher specificity; however, substantial heterogeneity was observed. Deep learning and multimodal approaches generally showed more favorable performance than traditional machine learning models, whereas large language model–based approaches showed more uncertain performance.

**Conclusions:**

AI models may have potential for identifying the PGS from cephalometric and hand–wrist radiographs and may support the timing of growth-modifying orthodontic interventions. However, substantial heterogeneity, limited external validation, and methodological weaknesses reduce the certainty and generalizability of current evidence. Multicenter studies are needed before routine clinical implementation.

**Supplementary Information:**

The online version contains supplementary material available at 10.1186/s12903-026-08627-6.

## Introduction

The pubertal growth spurt (PGS), characterized by peak height velocity (PHV), represents a critical period of bone remodeling and craniofacial development during which skeletal responsiveness to orthodontic and dentofacial orthopedic interventions is greatest [1]. Interventions aligned with this period are associated with superior orthopedic outcomes, whereas those initiated after the growth peak tend to yield diminished skeletal response and a greater risk of relapse [2, 3]. Because the onset and intensity of the PGS vary substantially among individuals, chronological age alone is an inadequate guide for clinical decision-making, and skeletal maturation assessment has become the standard approach for identifying pre-spurt, spurt, and post-spurt stages to guide treatment timing [4–8].

Among conventional two-dimensional (2D) radiographic methods, the Hand–Wrist Method (HWM) and the Cervical Vertebral Maturation (CVM) method are the most widely used in orthodontic practice. Fishman’s Skeletal Maturation Index (SMI) evaluates eleven skeletal maturity indicators derived from the ossification centers of the phalanges and carpal bones, with the PGS typically spanning SMI stages 3 to 6; its primary limitation is the requirement for an additional radiographic exposure beyond standard orthodontic records [5, 8, 9]. The CVM method, refined by Baccetti et al., classifies the morphology of vertebrae C2, C3, and C4 into six developmental stages (CS1–CS6), with the PGS corresponding to CS3–CS4, and can be assessed directly from the lateral cephalogram routinely obtained in clinical practice, thus eliminating supplementary radiation exposure [7, 10]. Although both methods demonstrate strong correlation with mandibular growth and PHV, their clinical application remains constrained by subjective visual interpretation and inter-observer variability, which can yield inconsistent diagnostic conclusions — particularly at the intermediate stages most critical to treatment planning [11, 12].

Artificial intelligence (AI) has emerged as a promising strategy to overcome these limitations. Machine learning (ML) encompasses algorithms that derive predictive rules from labeled data without task-specific programming, while deep learning (DL) employs multi-layered neural networks capable of extracting hierarchical feature representations directly from raw radiographic images, without manual feature engineering [13–15]. The methodological landscape in this field is broad: convolutional neural networks (CNNs) remain predominant and are well suited to spatial pattern recognition in radiographic data; Transformer-based and ensemble architectures have extended performance by integrating multi-regional anatomical information; classical ML approaches — including support vector machines, artificial neural networks, and stacking classifiers — remain applicable in smaller or more interpretable settings; and large language models (LLMs) have been evaluated for direct radiographic interpretation of hand–wrist images, albeit with variable recall across growth stages [13, 16–19]. In orthodontic practice, these models have been applied to cephalometric landmark detection, treatment planning, and the automated classification of CVM and HWM stages. More recently, multimodal and multi-stage architectures integrating data from different anatomical regions have been proposed to refine pubertal growth stage classification, representing a methodological advance over single-modality approaches [16].

Importantly, AI models in this field classify validated radiographic stages—such as CVM or SMI—as established proxies for the pubertal growth spurt, rather than predicting peak height velocity (PHV) directly. Therefore, the target outcome synthesized in this review should be interpreted as radiographic classification of maturation stages associated with the PGS, rather than direct biological measurement of growth velocity.

Despite these advances, the evidence base remains fragmented. Early reviews broadly addressed AI in orthodontic diagnostics—including anatomical segmentation, cephalometric landmark detection, and maturation stage classification—but were predominantly descriptive and lacked formal quantitative synthesis of diagnostic performance for PGS prediction. More recently, Kazimierczak et al. [20] reviewed 18 studies and reported accuracy values ranging from 57% to 95% for CVM-based models, highlighting substantial methodological heterogeneity and the absence of comparisons with hand–wrist methods. Sadeghi et al. [21] published the first quantitative meta-analysis on AI applied to CVM assessment, demonstrating globally promising model performance; however, their synthesis was restricted to cephalometric radiographs, did not incorporate hand–wrist studies, and did not systematically examine the influence of different AI architectures on diagnostic accuracy. Risk-of-bias assessments in both reviews relied on conventional diagnostic accuracy tools, without applying instruments specifically developed for AI-based studies, such as QUADAS-AI.

Consequently, three critical gaps persist in the literature: the absence of a comparative synthesis between AI models applied to cephalometric and hand–wrist radiographs for PGS prediction; the lack of systematic analysis of how AI architecture influences diagnostic performance; and the scarcity of rigorous methodological quality assessments using AI-specific tools.

The present systematic review and meta-analysis addresses these gaps by providing, to our knowledge, one of the first quantitative syntheses comparing AI diagnostic performance across both radiographic modalities, evaluating the contribution of different AI architectures to reported accuracy, and appraising methodological quality using QUADAS-AI. This integrated approach aims to offer a more comprehensive and clinically relevant evidence base to inform the development and implementation of AI-assisted skeletal maturation assessment in orthodontic practice.

## Methods

### Guidelines and registration

This systematic review and meta-analysis was conducted in accordance with the Preferred Reporting Items for Systematic Reviews and Meta-Analyses (PRISMA) 2020 guidelines [22]. The review protocol was prospectively registered in PROSPERO (CRD42024594040), an international database for systematic reviews. Methodological quality and risk of bias of the included studies were assessed using the QUADAS-AI (Quality Assessment of Diagnostic Accuracy Studies for Artificial Intelligence), a tool specifically designed for diagnostic accuracy studies employing AI models.

### Research question

The research question was structured using the PICO framework as follows:

- Population: children, adolescents, and young adults in the growth phase (≤ 21 years);
- Intervention: AI models applied to hand–wrist radiographs and/or lateral cephalometric radiographs as the primary imaging modality. Studies incorporating additional radiographic views (e.g., panoramic radiographs) were eligible provided that at least one of the two primary modalities was included and constituted the basis for skeletal maturation assessment;
- Comparator: traditional methods for skeletal maturation assessment (e.g., Fishman Index, Cervical Vertebral Maturation – CVM, or other validated standards);
- Outcome: model performance metrics (accuracy, sensitivity, specificity, area under the ROC curve – AUC, and F1-score), focusing on the identification of the pubertal growth spurt (PGS).

### Information sources and search strategy

A comprehensive literature search was last conducted until December 2025 across four electronic databases: PubMed (*n* = 213), Embase (*n* = 71), Web of Science (*n* = 258), and LILACS (*n* = 2), yielding a total of 544 records. The search was performed without restrictions on publication year or language to ensure a broad identification of records. However, for the eligibility phase, only original articles published in English, Portuguese, or Spanish were considered.

Search strategies combined controlled descriptors (MeSH, Emtree, DeCS) and free-text terms related to AI, machine learning, skeletal maturation, and cephalometric and/or hand–wrist radiographs. The complete search strings for each database are detailed in Supplementary Table S1. References were managed in Rayyan software (Qatar Computing Research Institute, Doha, Qatar) for duplicate removal and initial screening, and exported to Zotero (version 6.0) for full-text management.

### Eligibility criteria

#### Inclusion criteria

Studies were included if they met all the following requirements:

(1) assessed individuals aged ≤ 21 years; (2) employed AI models (machine learning, deep learning, or convolutional neural networks); (3) used lateral cephalometric and/or hand–wrist radiographs as the primary imaging modality; (4) predicted the PGS or classified validated skeletal maturation stages associated with it; (5) reported clear performance metrics (e.g., accuracy, sensitivity, specificity, AUC, or F1-score); and (6) were available in full text in English, Portuguese, or Spanish.

#### Exclusion criteria

Studies were excluded if they:

(1) predicted only chronological age without direct association with PGS; (2) focused exclusively on anatomical segmentation or image processing without functional prediction; (3) included populations with growth disorders, endocrine diseases, genetic syndromes, fractures, or other pathological bone conditions; (4) were reviews, conference abstracts, editorials, or unpublished manuscripts; and (5) did not report clear performance outcomes.

### Study selection

Study selection was performed independently and in duplicate by two reviewers (J.S.C. and M.L.R.), a dentist and a medical student, both with extensive experience in systematic review methodology and skeletal maturation assessment. The process occurred in two distinct stages:

- Stage 1 — Title and Abstract Screening: Of the 544 records identified, 351 unique records remained after removing duplicates (193 duplicates removed). During this stage, 175 records were excluded for not meeting the PICO criteria based on title and abstract review.
- Stage 2 — Full-Text Assessment: The remaining 176 records were assessed for eligibility through detailed full-text reading. Of these, 155 were excluded for the following reasons: age not eligible (*n* = 4); non-eligible language (*n* = 3); different imaging modality (*n* = 3); did not use AI (*n* = 1); specific clinical population (*n* = 11); prediction unrelated to pubertal growth spurt (*n* = 111); segmentation or processing only (*n* = 6); and full text unavailable (*n* = 16). A total of 21 studies were included in the final qualitative and quantitative synthesis.

**Fig. 1:**
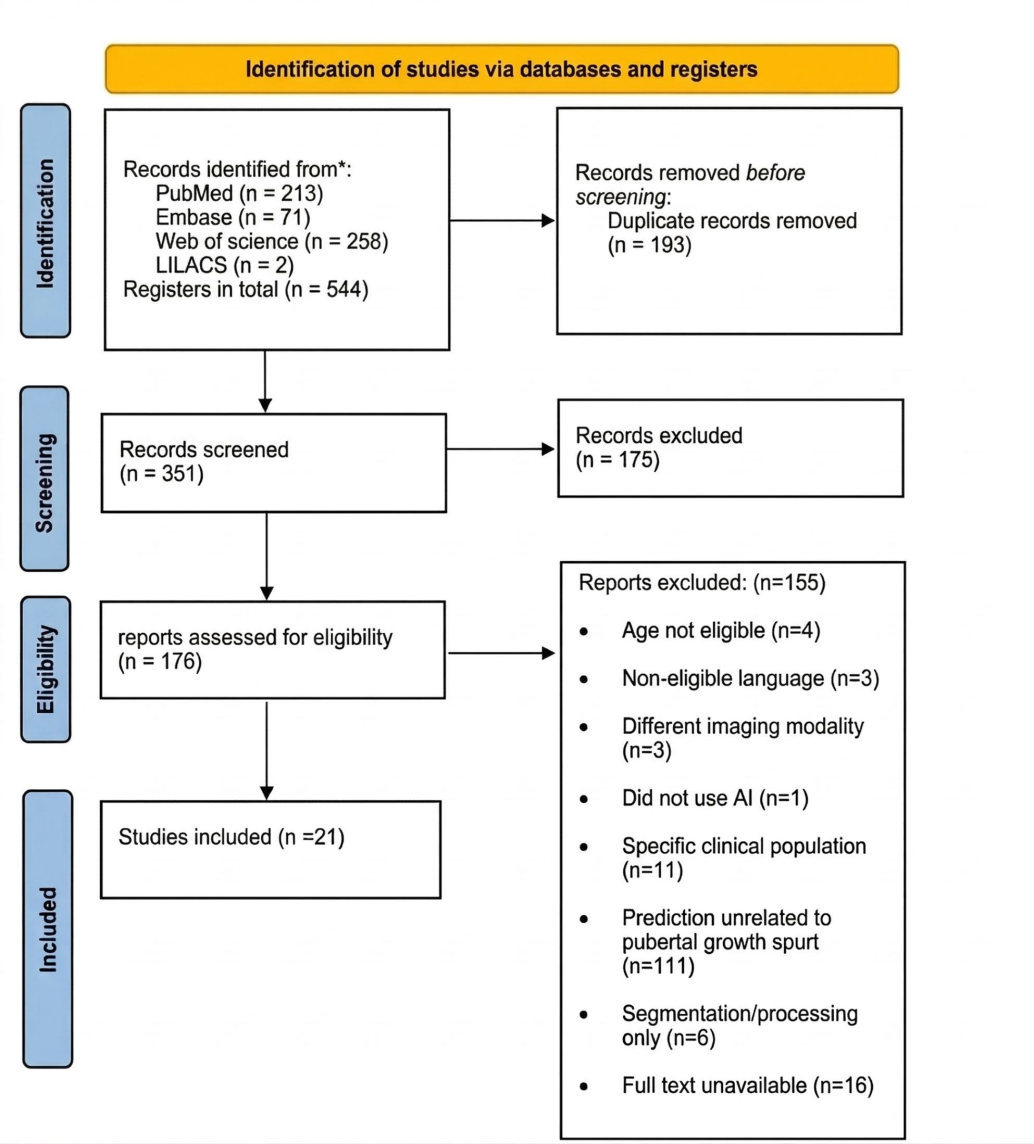
PRISMA 2020 flow diagram of study selection process

Disagreements at either stage were resolved through consensus meetings between the two reviewers. When consensus could not be reached, a third senior reviewer (J.A.D.) was consulted. The complete selection flow is detailed in the PRISMA 2020 flow diagram (Fig. 1).

### Data extraction and statistical analysis

The characteristics and outcomes of the included studies were summarized in structured tables. Data extraction was independently performed by trained reviewers using a standardized data extraction form to collect information on study design, sample distribution, imaging modality, reference standard, AI model type, validation approach, and diagnostic performance metrics. Individual study results were tabulated, and quantitative syntheses were visually displayed using forest plots to enable direct comparison across AI architectures and imaging modalities.

The evaluation of diagnostic performance was conducted through the systematic extraction or reconstruction of confusion matrices from all included studies. Each primary study frequently reported more than one predictive model or more than one imaging modality; to ensure comparability and avoid overweighting individual studies with multiple correlated estimates, only one model per study was selected for meta-analysis. When more than one eligible model was available, the best-performing model evaluated on the study’s test set was selected according to the primary performance metric reported by the authors, usually accuracy or AUC when available. This approach may have introduced optimism bias and should be considered when interpreting pooled estimates.

When studies explicitly reported confusion matrices already structured according to the three clinically relevant classes—pre-spurt, spurt, and post-spurt—data were directly extracted. In studies presenting results separately for different imaging modalities (e.g., hand–wrist and cervical vertebrae), the modality and model with the highest reported performance were selected for extraction.

For studies based on cervical vertebral maturation (CVM) using the six-stage classification (CS1–CS6), stages were harmonized into a three-class framework to ensure comparability across studies. Specifically, CS1 and CS2 were grouped as pre-spurt, CS3 and CS4 as spurt, and CS5 and CS6 as post-spurt. Confusion matrices were then reconstructed accordingly.

For studies based on hand–wrist radiographs, the classification systems varied substantially (e.g., Fishman Skeletal Maturation Index [SMI], Grave and Brown, DRU method), with differences in both the number of stages and the definition of pubertal phases. Therefore, the extraction and harmonization of confusion matrices for these studies were performed individually, based on the definitions provided by each study to approximate the three clinically relevant categories (pre-, peak-, and post-spurt) as closely as possible. For example, in studies using the Fishman Skeletal Maturation Index (SMI), stages SMI 1–3 were considered pre-spurt, SMI 4–6 as spurt (with SMI 6 corresponding to peak growth), and SMI 7–11 as post-spurt.

From each study, confusion matrices were used to obtain the fundamental diagnostic components: true positives (TP), false positives (FP), true negatives (TN), and false negatives (FN). These values served as the basis for calculating diagnostic performance metrics, including accuracy, sensitivity, specificity, precision, and F1 score, with corresponding 95% confidence intervals when applicable.

Pooled estimates of diagnostic performance were calculated using random-effects models to account for between-study heterogeneity. Sensitivity and specificity were synthesized using a bivariate random-effects model, which jointly accounts for the correlation between these parameters and between-study variability.

To further characterize diagnostic performance, hierarchical summary receiver operating characteristic (HSROC) models were applied. HSROC modeling allows for the joint estimation of sensitivity and specificity while accounting for potential threshold effects across studies. The model provides estimates of accuracy (θ), threshold (λ), and shape (β), enabling assessment of both central tendency and variability of diagnostic performance. HSROC curves were generated to visualize summary operating points, confidence regions, and prediction regions.

Between-study heterogeneity was assessed through visual inspection of forest plots, I² estimates for pooled accuracy, sensitivity, and specificity, and examination of prediction intervals. Heterogeneity was interpreted in light of variability in study design, imaging modality, reference standard, validation strategy, and AI architecture. Publication bias was explored through funnel plots of logit-transformed accuracy values, with qualitative assessment of asymmetry. Potential outliers were identified and their influence on pooled estimates was considered descriptively. Calibration metrics were not synthesized because they were inconsistently reported or unavailable in the included studies.

Exploratory meta-regression analyses were performed to investigate potential sources of between-study heterogeneity. The dependent variable was logit-transformed accuracy, and random-effects meta-regression models were fitted using restricted maximum likelihood estimation. The following study-level moderators were evaluated separately because of the limited number of included studies: imaging modality, AI architecture, sample size, log-transformed sample size, study design, and publication year. Results were interpreted cautiously due to the exploratory nature of the analysis and the small number of studies within some categories.

All statistical analyses were performed using R (version 4.5.0), with the MADA (Meta-Analysis of Diagnostic Accuracy) package for bivariate modeling. HSROC analyses and graphical outputs were additionally generated using OpenMeta [Analyst] (version 5.26.14).

### Risk of bias assessment

Methodological quality and risk of bias were evaluated using the QUADAS-AI tool, suitable for studies applying AI models to diagnostic tests. This tool assesses four main domains: (D1) Patient Selection, (D2) Index Test, (D3) Reference Standard, and (D4) Flow and Timing. Each domain was classified as having low, unclear, or high risk of bias. Two reviewers independently applied the tool, and discrepancies were resolved by consensus. The results were summarized in both tabular and graphical formats.

## Results

A total of 21 studies published between 2020 and 2025 were included in the quantitative synthesis [23–43], encompassing a wide range of imaging modalities, reference standards, and artificial intelligence approaches for predicting pubertal growth spurt. Study characteristics are summarized in Table 1, and the main quantitative findings are presented in Table 2.

**Table 1:** General characteristics of the included studies

Author (Year)	Country	Study Design	Sample Distribution (train/validation/test) – Validation method	Age range (years)	Imaging modality	Reference standard / Number of stages	AI type and best model	Ground-truth assessor	Main classification objective	Comparison with human experts
Akpınar and Salmanpour (2025) [23]	Turkey	Retrospective study	238 wrist + 238 cephalometric images	7–18	Hand–wrist + lateral cephalogram	CVS vertebrae	LLM (ChatGPT-4.0 iterative prompt)	3 experienced orthodontists	Evaluate ChatGPT accuracy for skeletal maturation	Inferior to human experts
Gonca et al. (2024) [24]	Turkey	Retrospective	1,067 (716/136/215)	7–18	Hand–wrist	Fishman SMI / 3 stages	MLP (supervised)	One orthodontist	Classify growth velocity	NR
Jiang et al. (2025) [25]	China	Retrospective multicenter	2100 (80/10/10 split)	6–19	Lateral cephalogram	QCVM (CS1–CS6)	CVNet (YOLOv3-based DL)	2 experienced orthodontists	Landmark detection + staging	Improved junior performance
Kanchanapiboon et al. (2024) [26]	Thailand	Retrospective cross-sectional	1380 (966/0/414)	4–21	Cephalometric	CVMS (CS1–CS6)	SVM (ML)	3 orthodontists (consensus)	Classify CVM stages	NR
Kavasoglu et al. (2025) [27]	Turkey	Retrospective	809 (10-fold CV)	10–18	Hand–wrist	Grave & Brown / 3 stages	Stacking ensemble (ML)	2 orthodontists + reviewer	Classify pubertal stages	Comparable to experts
Khazaei et al. (2023) [28]	Iran	Cross-sectional	1846 (1214/243)	5–18	Cephalometric	CVM (6 stages)	ConvNeXt (DL)	1 orthodontist	Classify pubertal phases	NR
Kim et al. (2021 a) [29]	South Korea	Retrospective	600 (480/120)	6–18	Cephalometric	CVM (CS1–CS6)	ResNet50 + U-Net	2 specialists	Classify CVM stages	NR
Kim et al. (2021 b) [30]	South Korea	Retrospective	499 (299/100/100)	6–18	Cephalometric	Fishman SMI	Ensemble regression	1 dentist + 1 orthodontist	Predict skeletal maturation	Yes
Kim et al. (2023) [31]	South Korea	Retrospective	3304 (2593/711)	6–18	Hand–wrist	Fishman SMI	Hybrid DL (Transformer + RetinaNet)	Orthodontists	Predict skeletal maturation	NR
Kök et al. (2020 a) [32]	Turkey	Retrospective	360 (70/30 split)	8–17	Cephalometric	CVM (6 stages)	ANN + Naive Bayes	1 orthodontist	Classify CVM stages	NR
Kök et al. (2021 b) [33]	Turkey	Retrospective	419 (70/15/15 split)	8–17	Cephalometric + wrist	Fishman SMI	ANN model	NA	Predict CVM stages	NR
Li et al. (2023) [34]	China	Retrospective	10,200 (7111/1544/1545)	NR	Cephalometric	CVM (CS1–CS6)	psc-CVM (YOLOv3 + DL)	Experienced orthodontists	Classify CVM stages	NR
Liu et al. (2024) [35]	China	Retrospective	7238 (CV + test)	9–19	Hand–wrist	DRU (7 stages)	Attention multi-task DL	Orthopedists + radiologists	Classify skeletal maturity	NR
Mohammed et al. (2024) [36]	Iraq	Retrospective	2400 (1920/480)	8–16	Cephalometric + panoramic	CVM + Demirjian	CNN (custom)	NA	Classify maturation stages	NR
Mohammad-Rahimi et al. (2022) [37]	Iran	Cross-sectional	890 (692/99/99)	NR	Cephalometric	CVM (CS1–CS6)	ResNet-101 DL	2 orthodontists	Classify maturation stages	Yes
Nogueira-Reis et al. (2024) [38]	Brazil	Retrospective + cross-sectional	600 (480/20/100)	6–17	Cephalometric	CVM (CS1–CS6)	Inception-v3 CNN	3 radiologists	Predict pubertal growth spurt	NR
Ramnarayan BK et al. (2025) [39]	India	Retrospective	525 (420/105)	7–17	Cephalometric	CVM (CVS1–CVS6)	VGG19 CNN	2 evaluators	Classify CVM stages	High agreement
Shoari et al. (2024) [40]	Iran	Retrospective cohort	663 (453/114/96)	8–15	Cephalometric	Mandibular growth rate	ResNet-18 CNN	Longitudinal growth	Classify growth stages	No
Tentaş and Özden (2025) [41]	Turkey	Retrospective	6572 (80/10/10 split)	8–16	Hand–wrist	Multiple staging systems	YOLOv8 DL	1 orthodontist	Classify skeletal maturation	High agreement
Yıldırım et al. (2025) [42]	Turkey	Retrospective comparative	90 images	8–16	Hand–wrist	Fishman + Greulich-Pyle	LLM (GPT models)	Experts	Predict bone age + stage	Inferior to experts
Zhang et al. (2024) [43]	China	Cross-sectional	1732 (~ 1299/433)	6–17	Cephalometric + wrist	CVM + SMI	CDSNet DL	Experts	Classify growth stages	Yes

*Abbreviations*: *AI* artificial intelligence, *ANN* artificial neural network, *CNN* convolutional neural network, *CVM* cervical vertebral maturation, *CVS* cervical vertebral stages, *DL* deep learning, *LLM* large language model, *ML* machine learning, *MLP* multilayer perceptron, *SMI* skeletal maturity index, *SVM* support vector machine, *U-Net* U-shaped convolutional network, *YOLO* You Only Look Once

**Table 2:** Reported performance metrics and confusion matrices of the best-performing AI models

Author (Year)	GROWTH SPURT TP / FN / FP / TN	PRE-SPURT TP / FN / FP / TN	POST-SPURT TP / FN / FP / TN	Accuracy	Precision	Sensitivity (Recall)	AUC (ROC)	F1 Score
Akpınar and Salmanpour (2025) [23]	72 / 25 / 20 / 121	45 / 17 / 58 / 118	40 / 39 / 3 / 156	0.470–0.517	0.496–0.589	0.470–0.517	0.82–0.89	0.467–0.524
Gonca et al. (2024) [24]	91 / 4 / 9 / 111	35 / 0 / 0 / 180	76 / 9 / 4 / 126	0.953	0.95	0.953	0.975	0.951
Jiang et al. (2025) [25]	NA	NA	NA	0.695	NR	NR	NR	NR
Kanchanapiboon et al. (2024) [26]	35 / 10 / 8 / 84	42 / 3 / 5 / 87	42 / 5 / 5 / 85	0.774	NR	NR	NR	NR
Kavasoglu et al. (2025) [27]	34 / 6 / 8 / 72	34 / 6 / 6 / 74	37 / 3 / 1 / 79	0.875	0.878	0.875	0.96	0.876
Khazaei et al. (2023) [28]	40 / 25 / 11 / 130	51 / 9 / 24 / 122	78 / 3 / 2 / 123	0.820–0.934	NR	NR	NR	NR
Kim et al. (2021 a) [29]	27 / 13 / 11 / 69	35 / 5 / 9 / 71	34 / 6 / 4 / 76	0.625	NR	NR	NR	NR
Kim et al. (2021 b) [30]	115 / 10 / 31 / 343	257 / 20 / 7 / 215	86 / 11 / 3 / 399	0.39	NR	NR	NR	NR
Kim et al. (2023) [31]	275 / 6 / 15 / 413	155 / 3 / 5 / 546	258 / 12 / 1 / 438	0.772	NR	NR	NR	NR
Kök et al. (2020 a) [32]	35 / 0 / 2 / 71	39 / 2 / 0 / 67	32 / 0 / 0 / 76	0.95	1.00	1.00	NR	1.00
Kök et al. (2021 b) [33]	20 / 1 / 1 / 41	19 / 2 / 1 / 41	20 / 1 / 0 / 42	0.904	NR	NR	NR	NR
Li et al. (2023) [34]	404 / 110 / 100 / 931	458 / 65 / 62 / 960	473 / 35 / 48 / 989	0.704	NR	NR	0.94	NR
Liu et al. (2024) [35]	930 / 22 / 29 / 765	NA	765 / 29 / 22 / 930	0.908–0.943	0.903–0.938	0.924–0.946	NR	0.919–0.942
Mohammed et al. (2024) [36]	399 / 1 / 2 / 798	400 / 0 / 0 / 800	398 / 2 / 1 / 799	0.96–0.98	NR	NR	NR	0.91–0.93
Mohammad-Rahimi et al. (2022) [37]	9 / 22 / 2 / 66	13 / 2 / 9 / 75	53 / 0 / 13 / 33	0.828	NR	NR	NR	NR
Nogueira-Reis et al. (2024) [38]	31 / 20 / 15 / 34	21 / 10 / 14 / 55	10 / 8 / 9 / 73	0.74–0.80	0.40–0.62	0.40–0.62	NR	NR
Ramnarayan BK et al. (2025) [39]	29 / 10 / 0 / 66	20 / 0 / 5 / 80	46 / 0 / 5 / 54	0.86	0.90	0.95	NR	0.86
Shoari et al. (2024) [40]	28 / 4 / 7 / 57	25 / 7 / 3 / 61	31 / 1 / 2 / 62	0.875	NR	NR	NR	NR
Tentaş and Özden (2025) [41]	142 / 14 / 20 / 298	144 / 18 / 6 / 306	148 / 8 / 14 / 304	0.91	0.87	0.91	0.91	0.88
Yıldırım et al. (2025) [42]	14 / 19 / 16 / 41	22 / 8 / 21 / 39	11 / 3 / 19 / 57	0.522	NR	NR	NR	NR
Zhang et al. (2024) [44]	134 / 14 / 13 / 240	72 / 1 / 10 / 318	158 / 22 / 14 / 207	0.909	NR	NR	NR	NR

*Abbreviations*: *TP* true positives, *FP* false positives, *FN* false negatives, *TN* true negatives, *AUC* area under the curve, *NA* not applicable, *NR* not reported

### Qualitative synthesis

#### General characteristics of the included studies

Most included studies adopted a retrospective study design, while a smaller proportion utilized cross-sectional designs. Sample sizes varied substantially across studies, ranging from 90 images [42] to robust databases comprising over 10,200 radiographs [34]. The age range of the analyzed individuals spanned from 4 to 21 years, effectively capturing the critical period of skeletal growth and development. Geographically, the studies reflect a global effort, with notable concentrations of publications originating from Turkey, China, South Korea, and Iran.

#### Imaging modalities and reference standards

The primary imaging modalities investigated were lateral cephalometric radiographs and hand-wrist radiographs. Some studies adopted multimodal approaches, combining hand-wrist and cephalometric images, or even panoramic radiographs, to enhance predictive performance. To establish the ground truth, the studies predominantly relied on the visual assessment of human experts, such as experienced orthodontists and radiologists, occasionally operating under consensus. For cephalometric radiographs, the Cervical Vertebral Maturation (CVM) system (predominantly the six-stage CS1-CS6) was the most widely used. For hand-wrist analysis, classification systems varied, including the Fishman Skeletal Maturation Index (SMI), the Grave & Brown method, and the DRU method.

#### Artificial intelligence architectures

Considerable diversity was observed in the artificial intelligence architectures developed for growth classification. Deep Learning (DL) was the predominant approach, frequently employing Convolutional Neural Networks (CNNs)—such as ResNet, VGG, ConvNeXt, and Inception—as well as object detection architectures (e.g., YOLO) and attention-based models (Transformers). Traditional Machine Learning (ML) algorithms, including Support Vector Machines (SVM), Artificial Neural Networks (ANN), Multilayer Perceptrons (MLP), and Ensemble methods, were also successfully reported. More recently, approaches utilizing Large Language Models (LLMs), such as iterations of ChatGPT, have been tested for this purpose, although results indicate their performance remains inferior to human experts for the direct interpretation of maturation stages from radiographs.

#### Methodological quality and risk of bias

**Fig. 2:**
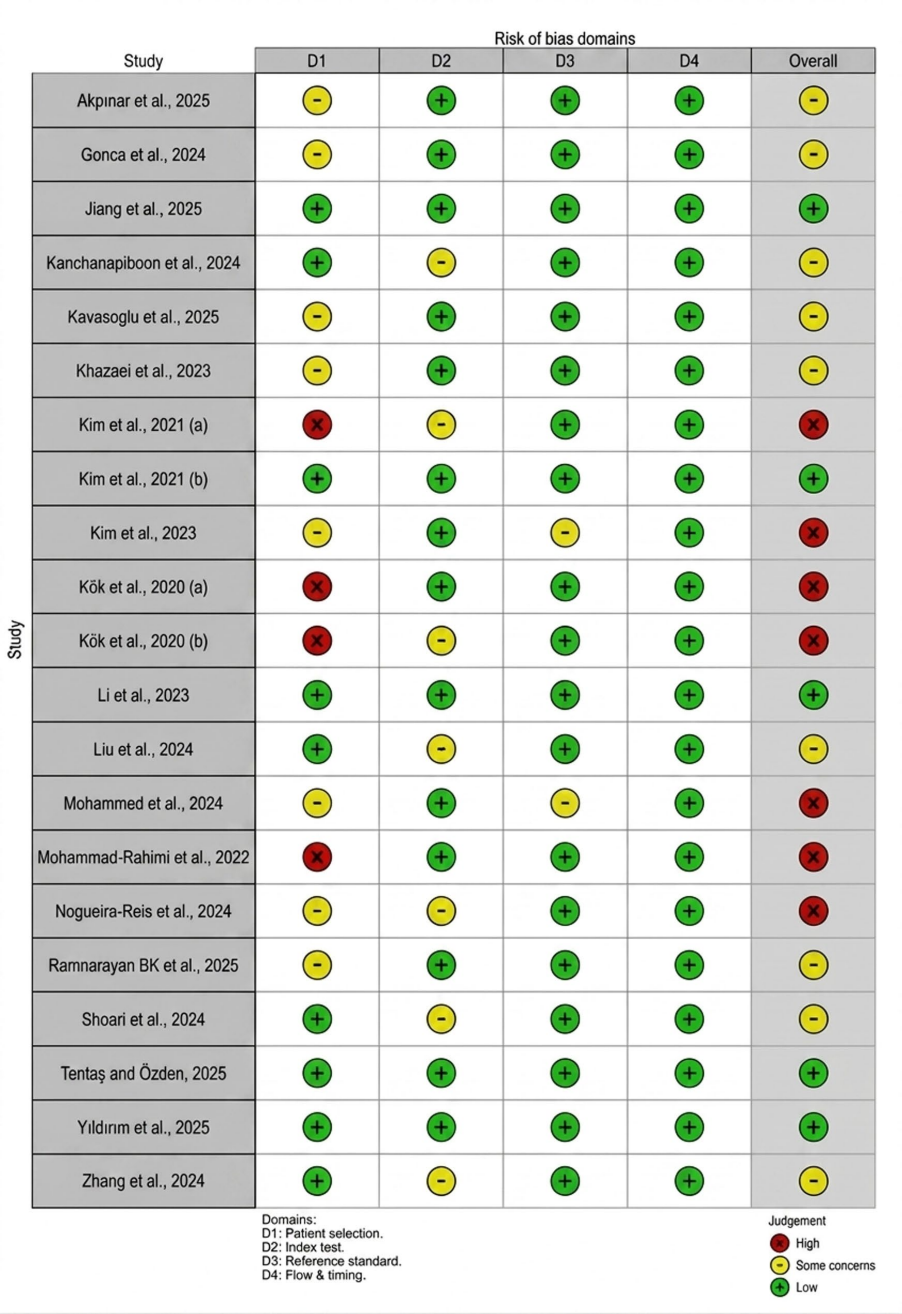
QUADAS-AI Risk of Bias Assessment

The risk-of-bias assessment indicated an overall moderate methodological quality with a considerable proportion of studies presenting some concerns or high risk. As shown in the bar summary (Supplementary Figure S6), the domains Reference Standard and Flow and Timing demonstrated consistently low risk of bias, with Flow and Timing rated as low risk across all studies. In contrast, Patient Selection showed the greatest variability, including several studies classified as high risk, mainly due to insufficient reporting of sampling strategies. The Index Test domain also presented some concerns, although most studies were rated as low risk. As illustrated in Fig. 2, only a minority of studies were classified as low risk across all domains, while the majority exhibited moderate risk (some concerns) or high risk of bias, particularly driven by issues in patient selection and, to a lesser extent, index test methodology.

### Quantitative synthesis

Across studies, model performance was generally high but heterogeneous. Reported accuracies ranged from 0.39 [30] to 0.98 [36], reflecting substantial variability in datasets, methodological design, and model architectures. The pooled accuracy, including 21 studies [23–43], from the random-effects model was 0.83 (95% CI: 0.76–0.89), indicating overall good performance. However, heterogeneity was considerable (I² = 97.97%), and the wide prediction interval (0.37–0.98) suggests that the performance of future studies may vary markedly depending on context.

**Fig. 3:**
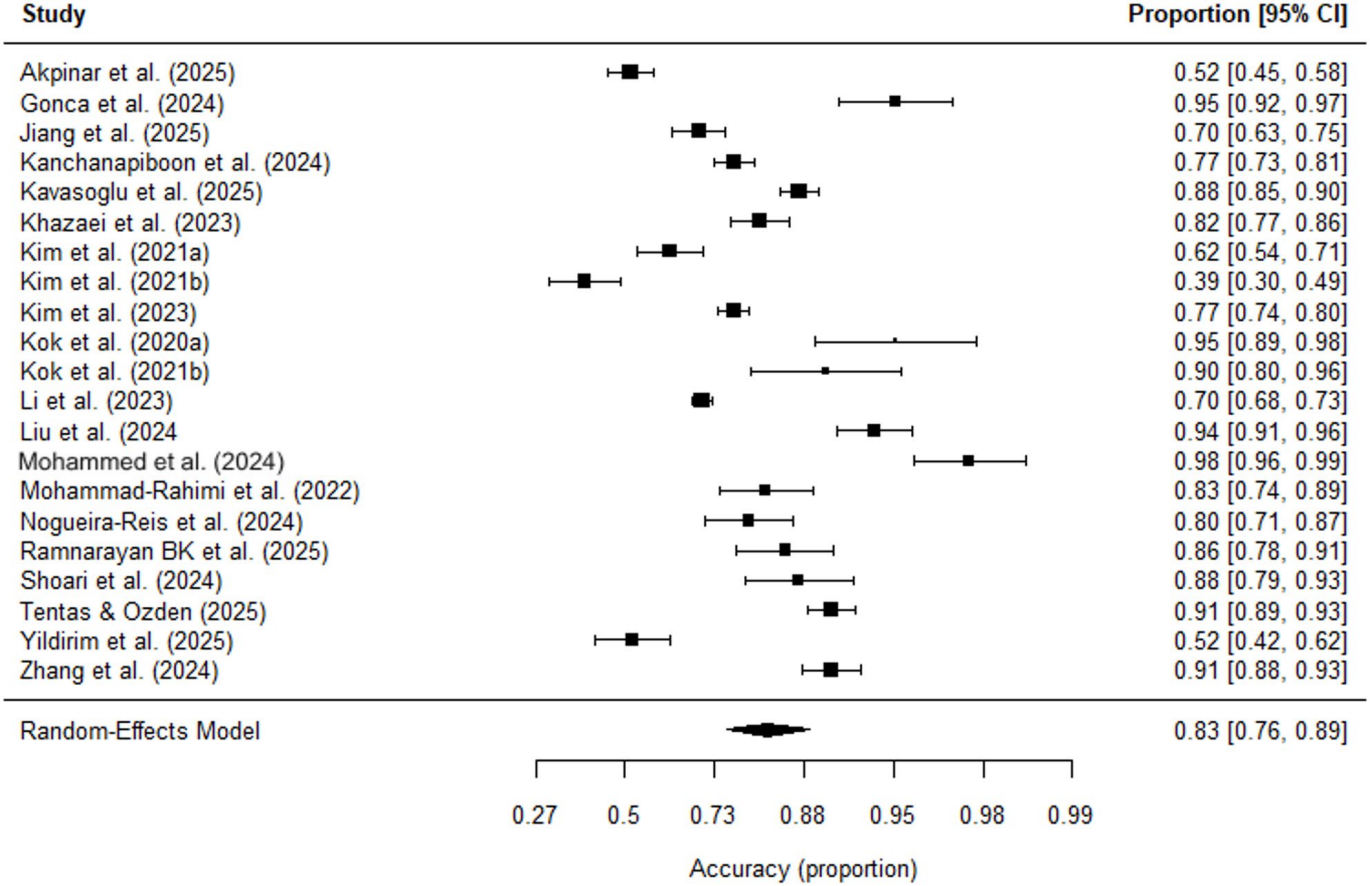
Forest plot of the accuracy of the best model for pubertal growth spurt prediction in the included studies

The distribution of accuracy estimates across studies is shown in Fig. 3. While several studies demonstrated high precision with narrow confidence intervals, others exhibited greater uncertainty, likely due to smaller sample sizes or differences in validation strategies.

**Fig. 4:**
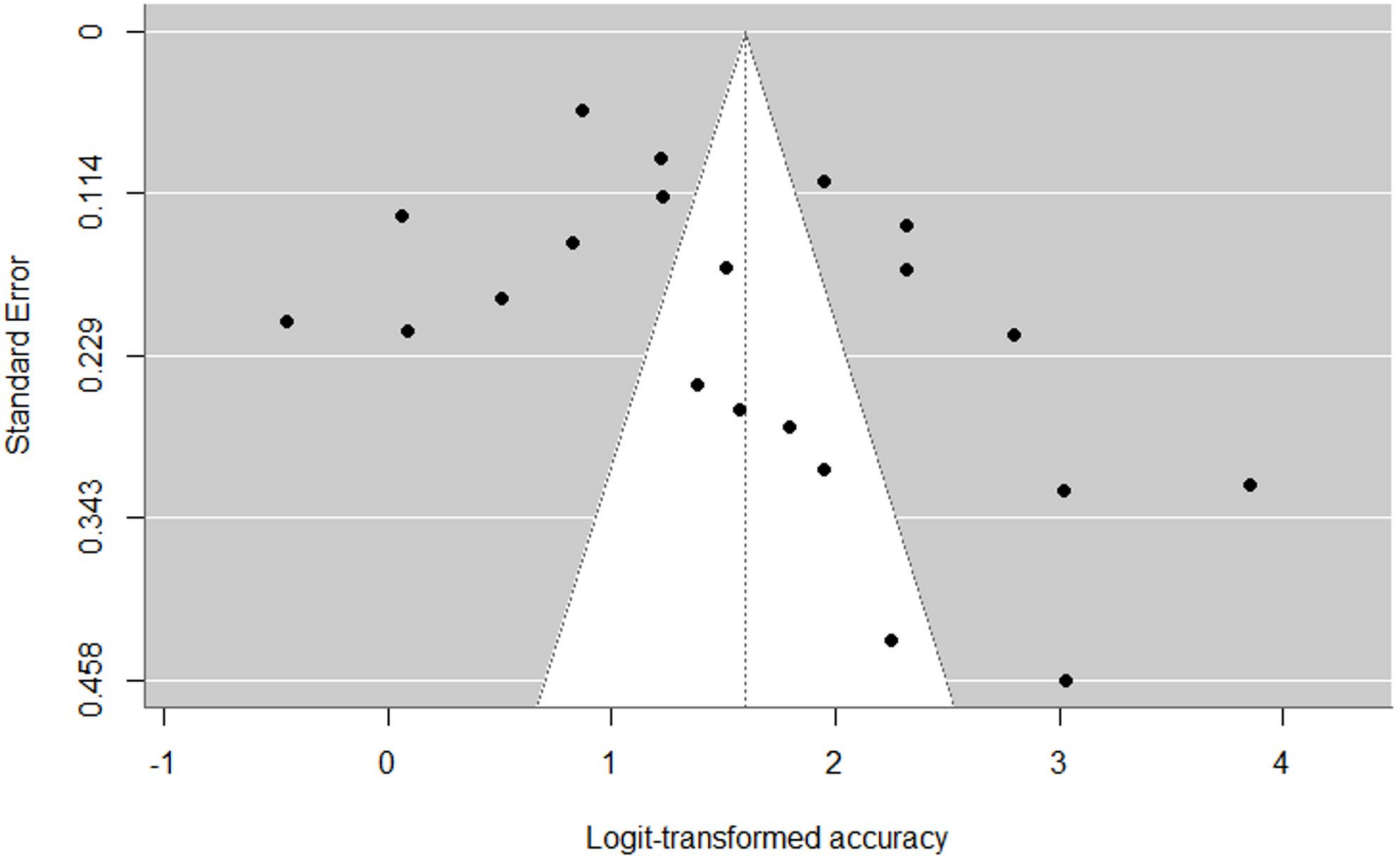
Funnel Plot for Assessment of Publication Bias

Visual inspection of the funnel plot (Fig. 4) revealed asymmetry, with a predominance of studies reporting higher accuracy values and fewer studies on the lower-performance side. Although this pattern may suggest small-study effects or potential publication bias, the substantial between-study heterogeneity indicates that asymmetry may also reflect genuine differences in study design, populations, imaging modalities, and model types. Therefore, because publication bias was assessed qualitatively and in the presence of marked heterogeneity, these findings should be interpreted cautiously and cannot definitively establish or exclude publication bias.

**Fig. 5:**
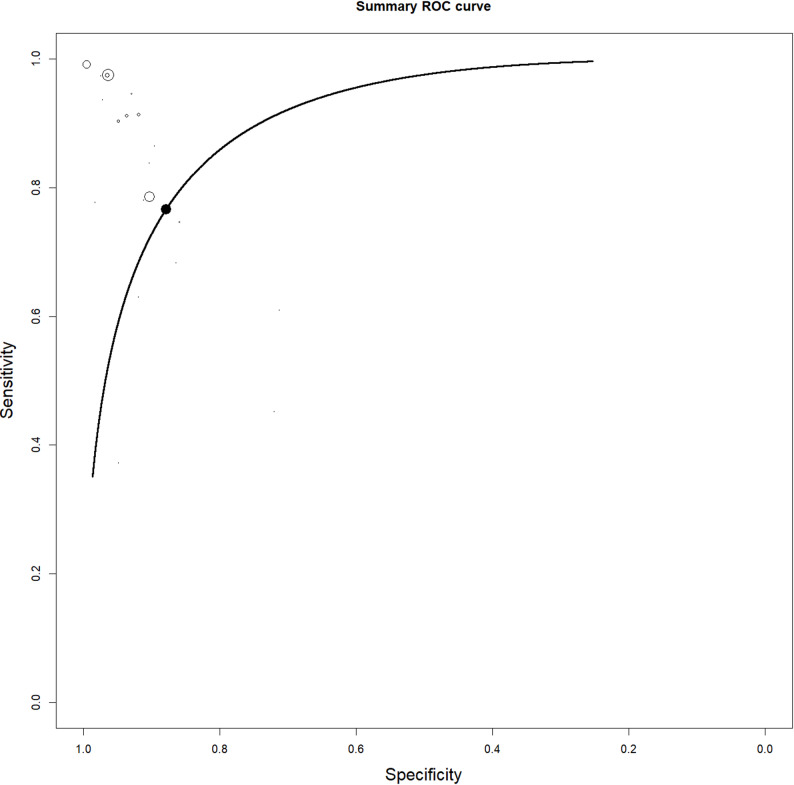
Summary ROC curve for pubertal growth spurt prediction

Diagnostic performance was further explored using HSROC models. Overall, the HSROC curve (Fig. 5), including 20 studies [23, 24, 26–43], demonstrated good discriminative ability. The accuracy parameter (θ = 0.23, 95% HPD: −0.01 to 0.42) indicated discrimination above chance, although with some uncertainty. The threshold parameter (λ = 1.91) suggested a specificity-oriented operating point, and no evidence of a threshold effect was observed (β ≈ 0). Summary estimates yielded a sensitivity of 0.76 (95% HPD: 0.70–0.82) and specificity of 0.88 (95% HPD: 0.83–0.91). However, wide prediction intervals highlighted substantial variability across settings.

**Fig. 6:**
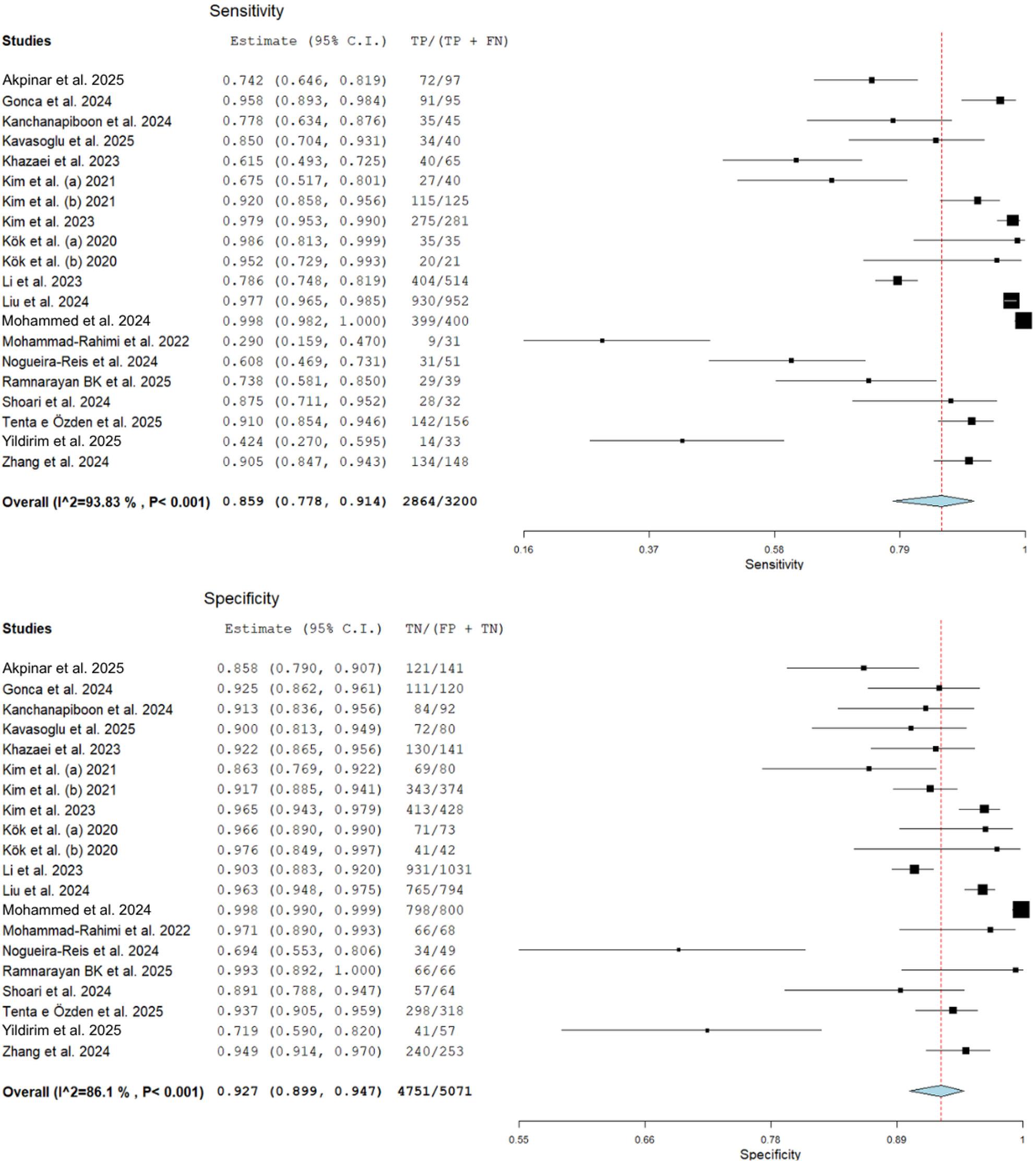
Diagnostic Sensitivity and Specificity for Predicting Pubertal Growth Spurt

Complementary pooled estimates based on bivariate random-effects models (Fig. 6) showed a sensitivity of 0.86 (95% CI: 0.78–0.91) and specificity of 0.93 (95% CI: 0.90–0.95), confirming strong diagnostic performance despite persistent heterogeneity (I² > 85% for both parameters).

**Fig. 7:**
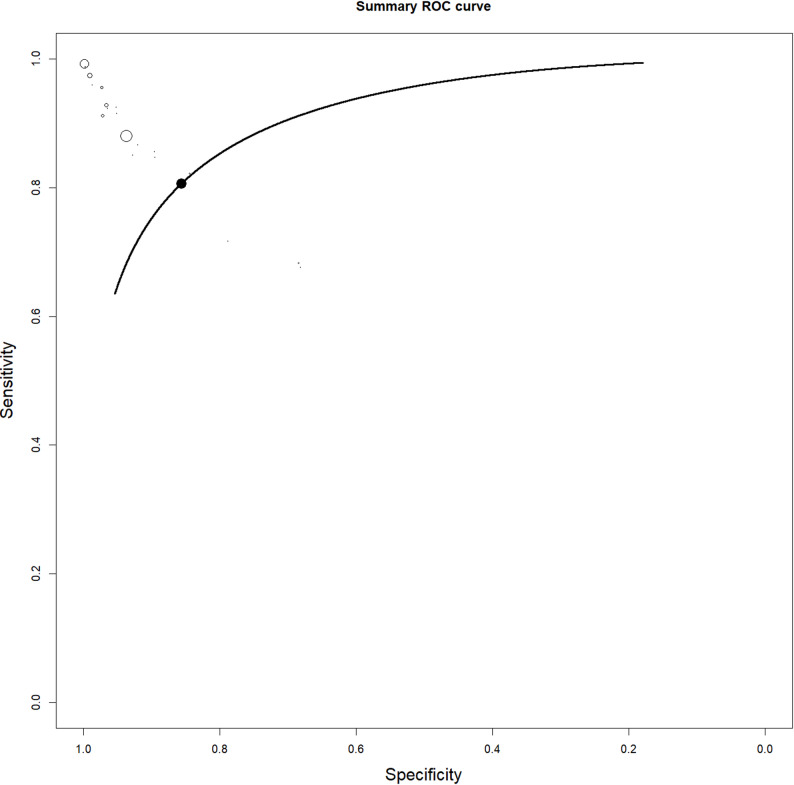
HSROC curve for prediction of the pubertal pre-spurt stage

Subgroup analyses by growth phase revealed distinct patterns. For the pre-spurt stage (Fig. 7), including 19 studies [23, 24, 26–33, 35–43], discrimination was limited (θ = 0.01, 95% HPD: −0.14 to 0.16), with heterogeneity primarily driven by variability in threshold selection rather than intrinsic test performance. Summary estimates showed sensitivity of 0.80 (95% HPD: 0.76–0.83) and specificity of 0.85 (95% HPD: 0.81–0.88), although prediction intervals remained wide.

**Fig. 8:**
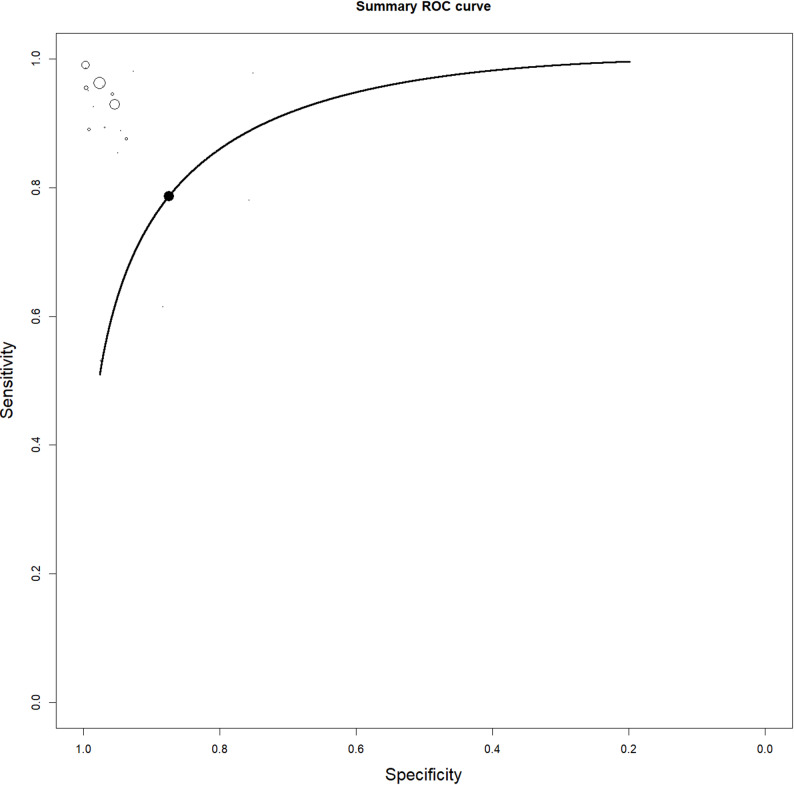
HSROC curve for post-spurt stage prediction

For post-spurt prediction (Fig. 8), including 20 studies [23, 24, 26–43], discrimination was modest but slightly more consistent (θ = 0.14, 95% HPD: −0.16 to 0.40). Both threshold variability (σᵅ = 1.70) and accuracy variability (σθ = 0.39) contributed to heterogeneity. Sensitivity was 0.78 (95% HPD: 0.71–0.85) and specificity 0.87 (95% HPD: 0.81–0.92), with similarly broad prediction intervals.

Subgroup analyses according to imaging modality and algorithm type are presented in Supplementary Figures S1–S5. Studies based on cervical vertebral maturation (CVM) (Supplementary Figure S1), including 15 studies [23, 25, 26, 28–30, 33, 34, 36–40, 43], showed discrimination above chance (θ = 0.34, 95% HPD: 0.05–0.76), with sensitivity of 0.72 (95% HPD: 0.59–0.81) and specificity of 0.89 (95% HPD: 0.83–0.93). In contrast, hand–wrist-based studies (Supplementary Figure S2), including 6 studies [24, 27, 31, 35, 41, 42], showed higher sensitivity, 0.78 (95% HPD: 0.63–0.88), but lower specificity, 0.82 (95% HPD: 0.69–0.88), with a more uncertain accuracy parameter (θ = 0.13). Thus, subgroup analysis suggested a sensitivity advantage for hand–wrist-based models and a specificity advantage for cephalometric-based models, although heterogeneity remained substantial and confidence intervals overlapped.

When stratified by algorithm type, deep learning models (Supplementary Figure S3), including 13 studies [25, 28, 29, 31–40, 43], achieved balanced performance (sensitivity 0.72; specificity 0.88) with consistent discrimination (θ = 0.35). Traditional machine learning algorithms (Supplementary Figure S4), including 5 studies [24, 26, 27, 32, 33], showed slightly higher sensitivity, 0.76, but lower specificity, 0.84, with greater variability. In contrast, large language model (LLM)-based approaches (Supplementary Figure S5), including 2 studies [23, 42], demonstrated the most uncertain performance, with wide intervals for both sensitivity (0.57) and specificity (0.78).

These findings suggest possible performance differences across AI architectures, with deep learning models showing more balanced performance, traditional ML models tending toward higher sensitivity and lower specificity, and LLM-based approaches showing the lowest stability across studies. However, these subgroup findings should be interpreted cautiously because of the small number of studies in some categories and the indirect nature of subgroup comparisons.

Across all subgroup analyses, heterogeneity remained substantial, with wide prediction intervals indicating marked variability in performance across settings. To further explore potential sources of heterogeneity, exploratory random-effects meta-regression analyses were performed using logit-transformed accuracy as the dependent variable. Imaging modality was not significantly associated with accuracy (QM = 1.67, *p* = 0.435), nor were sample size (QM = 0.95, *p* = 0.329), log-transformed sample size (QM = 0.51, *p* = 0.477), study design (QM = 0.30, *p* = 0.859), or publication year (QM = 0.02, *p* = 0.898). AI architecture showed a non-significant trend toward explaining heterogeneity (QM = 5.19, *p* = 0.075), accounting for approximately 14.2% of between-study variance. In this model, LLM-based approaches showed lower accuracy than deep learning models, although this finding should be interpreted cautiously because only two studies evaluated LLM-based approaches. Residual heterogeneity remained substantial across all models.

Overall, AI-based models demonstrated good average discriminatory performance for predicting the pubertal growth spurt; however, performance varied considerably across studies. Differences in imaging modality, reference standard, study design, validation strategy, and algorithm choice appear to be important drivers of heterogeneity and should be considered when interpreting these findings.

## Discussion

The findings of this systematic review and meta-analysis indicate that AI models demonstrate overall good performance in predicting the PGS, with a pooled accuracy of 0.83 (95% CI: 0.76–0.89) and favorable sensitivity and specificity estimates derived from hierarchical models. However, these results should be interpreted cautiously, given the considerable heterogeneity observed (I² ≈ 98%) and the wide prediction intervals, which indicate that performance may vary markedly depending on the clinical and methodological context.

Importantly, although several studies reported high values of sensitivity, specificity, and AUC, these metrics were derived from heterogeneous thresholds, reference standards, and classification schemes. Therefore, they do not represent a fully comparable or standardized diagnostic accuracy framework. As such, the present synthesis does not support definitive conclusions regarding diagnostic performance, but rather reflects a descriptive aggregation of available evidence. Accordingly, AI should be considered a promising and evolving tool rather than a validated substitute for established clinical assessment methods.

The variability in model performance is likely multifactorial. Important sources of heterogeneity include differences in imaging modality, reference standards (CVM, SMI, DRU, and mandibular growth rate), sample composition, age range, population characteristics, dataset size, and validation strategy. In particular, the use of different maturation systems may have had a major influence on the pooled estimates, because these systems capture related but not identical aspects of skeletal development. Likewise, differences in training/test splits, internal cross-validation approaches, and the rarity of external validation may have further amplified performance variability across studies. In addition, inconsistencies in how developmental stages were defined and grouped—particularly when collapsing multistage systems such as CS1–CS6 or SMI 0–11 into broader clinical categories—further limit direct comparability across studies.

Subgroup analyses provided additional insights. Hand–wrist-based models showed numerically higher sensitivity, whereas cephalometric-based approaches showed higher specificity, suggesting potentially complementary strengths between modalities. Similarly, deep learning models appeared more stable than traditional machine learning approaches, whereas LLM-based models showed the greatest uncertainty. Exploratory meta-regression analyses corroborated these patterns, with AI architecture showing a non-significant trend toward explaining heterogeneity (*p* = 0.075, R² ≈ 14.2%), while imaging modality, sample size, study design, and publication year were not significantly associated with accuracy. However, these findings should be interpreted cautiously, as they were based on indirect comparisons with persistent residual heterogeneity and a small number of studies in some subgroups.

When contextualized within the existing literature, the present findings are broadly consistent with previous systematic reviews, such as those by Kazimierczak et al. (2024) [20] and Sadeghi et al. (2025) [21], which also reported high but variable performance of AI models for skeletal maturation assessment. However, by incorporating multimodal approaches and a formal quantitative synthesis, this study expands upon previous work and highlights the extent to which methodological variability influences reported performance metrics.

A critical methodological concern identified across the included studies is the limited use of robust validation strategies. Although many studies reported high accuracy values, external validation was rarely performed, and internal validation methods were often insufficiently described. This raises the possibility of overfitting, particularly in studies using small or highly curated datasets. Models trained and tested on similar data distributions may capture dataset-specific patterns rather than generalizable features, leading to overestimation of performance. This concern is consistent with broader evidence from radiologic AI, in which performance frequently declines when models are tested in independent external datasets [44]. Consequently, the reproducibility and clinical applicability of these models in independent populations remain uncertain.

A central review-level limitation concerns the selection of only the best-performing model from each study for inclusion in the meta-analysis. Although this approach was adopted to avoid double-counting correlated estimates from the same study, it may have introduced optimism bias and led to an overestimation of real-world diagnostic performance. In diagnostic AI research, selecting the highest-performing model within each study may produce summary estimates that more closely represent an upper-bound of model performance under favorable experimental conditions than the average performance expected in routine clinical settings. Accordingly, the pooled accuracy of 0.83 (95% CI: 0.76–0.89) should be interpreted cautiously and should not be considered a direct estimate of performance in unselected clinical populations. Similarly, the harmonization of heterogeneous skeletal maturation systems into a three-class framework and the reconstruction of confusion matrices, although necessary for quantitative synthesis, may have introduced misclassification bias or loss of information, particularly for studies using hand–wrist classification systems with distinct staging definitions.

The inclusion of participants up to 21 years of age also warrants careful consideration. This upper age limit was adopted to capture the full spectrum of skeletal maturation, including the post-pubertal stabilization phase, which is relevant for distinguishing post-spurt from active growth stages. However, most individuals in late adolescence and early adulthood are biologically post-pubertal, particularly females. Their inclusion may therefore have contributed to inflated classification performance by increasing the proportion of morphologically clear post-spurt cases, while reducing uncertainty in a category that is generally easier to classify than the clinically critical pre-peak and peak phases. Nevertheless, this criterion also reflects a relevant clinical scenario, as orthodontic decision-making in late adolescence may require confirmation that active growth has ceased. Future studies should provide age-stratified analyses and focus more specifically on the diagnostically challenging pre-peak and peak pubertal phases, in which accurate classification is most relevant for treatment timing.

From a clinical perspective, AI-based tools may have potential as adjunctive instruments to support the assessment of skeletal maturation and the timing of orthodontic interventions. Their use could contribute to reducing subjectivity, improving workflow efficiency, and enhancing reproducibility, particularly in high-demand or resource-limited settings. However, given the current limitations in validation, standardization, and interpretability, these models should not be considered replacements for expert clinical judgment or established reference methods. This more cautious role for AI as a support tool is in line with broader discussions on current clinical applications of AI in radiology [45].

Beyond diagnostic performance, clinical integration of AI in orthodontics also requires attention to ethical, legal, and data-governance issues. Radiographic AI systems depend on secure handling of patient data, appropriate anonymization procedures, and protection against data breaches or unauthorized reuse. In addition, given the limited interpretability of many algorithms and the absence of clear legal frameworks for autonomous decision-making in many settings, these systems should currently be positioned within a human-in-the-loop model, in which AI supports—but does not replace—professional judgment and responsibility.

Future research should prioritize the development of standardized protocols for image acquisition, staging systems, and outcome definitions, as well as the implementation of rigorous external validation in diverse populations. Multicenter datasets and prospective study designs will be essential to improve generalizability. In addition, the use of explainable AI approaches may enhance transparency and facilitate clinical integration, as interpretability remains a key challenge in medical imaging AI [46]. Finally, more robust diagnostic accuracy syntheses based on harmonized thresholds and definitions will be necessary to establish more reliable estimates of performance.

In summary, AI-based models show promising but variable performance in predicting the PGS. Although current evidence supports their potential as supportive tools in clinical practice, substantial methodological limitations and lack of standardization preclude definitive conclusions regarding their diagnostic accuracy or routine clinical implementation.

## Conclusion

In conclusion, AI-based models demonstrate promising but heterogeneous performance in predicting the PGS from cephalometric and hand–wrist radiographs. Although pooled estimates suggest good discriminative capacity, these values likely represent an upper-bound estimate of best-case performance rather than expected real-world accuracy, given the systematic selection of best-performing models, the predominance of internal validation, and the substantial methodological inconsistencies observed. Together with the limited external validation and between-study variability, these factors restrict the strength and generalizability of the current evidence. At present, AI should be regarded as a supportive tool rather than a substitute for established clinical assessment methods. Future research focusing on standardized methodologies, robust external validation frameworks, and multicenter prospective datasets will be essential to clarify the true clinical utility of these models and support their safe integration into orthodontic practice.

## Supplementary Information

Supplementary Material 1.

Supplementary Material 2.

Supplementary Material 3: Supplementary Table S1. Full electronic search strategies for all databases. Supplementary Figure S1 – HSROC curve for cervical vertebral maturation (CVM)-based studies. Supplementary Figure S2 – HSROC curve for hand–wrist-based studies. Supplementary Figure S3 – HSROC curve for deep learning models (CNN, Transformer, and YOLO). Supplementary Figure S4 – HSROC curve for traditional machine learning models (SVM, MLP, and ensemble methods). Supplementary Figure S5 – HSROC curve for large language model (LLM)-based approaches. Supplementary Figure S6 – Bar Plot of QUADAS-AI Summary Risk-of-Bias Ratings.

## Data Availability

All data supporting the findings of this study are contained within the article and its supplementary material. Additional details of the data extraction and analysis are available from the corresponding author on reasonable request.

## References

[1] Franchi L, Baccetti T, McNamara JA Jr. Mandibular growth as related to cervical vertebral maturation and body height. Am J Orthod Dentofac Orthop. 2000;118(3):335–40. 10.1067/mod.2000.107009 10982936

[2] Soliman A, De Sanctis V, Elalaily R, Bedair S. Advances in pubertal growth and factors influencing it: Can we increase pubertal growth? Indian J Endocrinol Metab. 2014;18(Suppl 1):S53–62. 25538878 10.4103/2230-8210.145075 PMC4266869

[3] Szemraj-Folmer A, Wojtaszek-Słomińska A, Racka-Pilszak B, Kuc-Michalska M. Assessment of the duration of the pubertal growth spurt in patients with skeletal open bite. J Orofac Orthop. 2021;82(2):92–8. 33306144 10.1007/s00056-020-00262-2 PMC7904551

[4] Ramírez-Velásquez M, Viloria-Ávila TJ, Rodríguez DA, Rojas ME, Zambrano O. Maturation of cervical vertebrae and chronological age in children and adolescents. Acta Odontol Latinoam. 2018;31(3):125–30. 30829366

[5] Fishman LS. Chronological versus skeletal age: An evaluation of craniofacial growth. Angle Orthod. 1979;49(3):181–9. 225970 10.1043/0003-3219(1979)049<0181:CVSAAE>2.0.CO;2

[6] Araujo MTS, Cury-Saramago AA, Motta AFJ. Guias clínicos e radiográficos utilizados para a predição do surto de crescimento puberal. Dent Press J Orthod. 2011;16(5):98–103.

[7] Baccetti T, Franchi L, McNamara JA Jr. The cervical vertebral maturation (CVM) method for the assessment of optimal treatment timing in dentofacial orthopedics. Semin Orthod. 2005;11(3):119–29.

[8] Fishman LS. Radiographic evaluation of skeletal maturation: A clinically oriented method based on hand-wrist films. Angle Orthod. 1982;52(2):88–112. 6980608 10.1043/0003-3219(1982)052<0088:REOSM>2.0.CO;2

[9] Fishman LS. Skeletal maturation indicators: The hand-wrist radiograph. Am J Orthod. 1982;81(5):439–42.

[10] McNamara JA Jr, Franchi L. The cervical vertebral maturation method: A user’s guide. Angle Orthod. 2018;88(2):133–43. 29337631 10.2319/111517-787.1 PMC8312535

[11] Nestman TS, Marshall SD, Qian F, et al. Cervical vertebrae maturation method morphologic criteria: poor reproducibility. Am J Orthod Dentofac Orthop. 2011;140(2):182–8. 10.1016/j.ajodo.2011.04.013 21803255

[12] Eninanç İ, Büyükbayraktar ZÇ. Assessment of correlation between hand-wrist maturation and cervical vertebral maturation: a fractal analysis study. BMC Oral Health. 2023;23(1):798. 37884998 10.1186/s12903-023-03483-0 PMC10601178

[13] Zhou J, Zhou H, Pu L, et al. Development of an artificial intelligence system for the automatic evaluation of cervical vertebral maturation status. Diagnostics (Basel). 2021;11(12):2200. 34943436 10.3390/diagnostics11122200 PMC8700528

[14] Zogala D. Artificial intelligence in medical imaging. Cas Lek Cesk. 2024;162(7–8):279–82. 38981712

[15] LeCun Y, Bengio Y, Hinton G. Deep learning. Nature. 2015;521:436–44. 26017442 10.1038/nature14539

[16] Motie P, Ashkan A, Mohammad-Rahimi H, Hassanzadeh-Samani S, Razzaghi N, Behnaz M, Shahab S, Motamadian SR. Improving cervical maturation degree classification accuracy using a multi-stage deep learning approach. Imaging Sci Dent. 2025;55(3):290–301. 10.5624/isd.20250045. 41070250 10.5624/isd.20250045 PMC12505443

[17] Cicek O, Özçelik YB, Altan A. A new approach based on metaheuristic optimization using chaotic functional connectivity matrices and fractal dimension analysis for AI-driven detection of orthodontic growth and development stage. Fractal Fract. 2025;9(3):148.

[18] Ozkan E, Koyun M. Atlas-Assisted Bone Age Estimation from Hand–Wrist Radiographs Using Multimodal Large Language Models: A Comparative Study. Diagnostics (Basel). 2026;16(3):487. 10.3390/diagnostics16030487. 41681804 10.3390/diagnostics16030487 PMC12896913

[19] Yıldırım A, Cicek O. Assessment of AI-driven large language models for orthodontic aesthetic scoring using the IOTN-AC. Diagnostics (Basel). 2025;15(23):3048. 10.3390/diagnostics15233048. 41374429 10.3390/diagnostics15233048 PMC12691508

[20] Kazimierczak W, Jedliński M, Issa J, Kazimierczak N, Janiszewska-Olszowska J, Dyszkiewicz-Konwińska M, Różyło-Kalinowska I, Serafin Z, Orhan K. Accuracy of artificial intelligence for cervical vertebral maturation assessment: a systematic review. J Clin Med. 2024;13(14):4047. 10.3390/jcm13144047. 39064087 10.3390/jcm13144047 PMC11277636

[21] Sadeghi TS, Ourang SA, Sohrabniya F, et al. Performance of artificial intelligence on cervical vertebral maturation assessment: a systematic review and meta-analysis. BMC Oral Health. 2025;25(1):187. 39910512 10.1186/s12903-025-05482-9 PMC11796225

[22] Page MJ, McKenzie JE, Bossuyt PM, Boutron I, Hoffmann TC, Mulrow CD et al. The PRISMA 2020 statement: An updated guideline for reporting systematic reviews. British Medical Journal. 2021;372(71). Available from: https://www.bmj.com/content/372/bmj.n71. 10.1136/bmj.n71 PMC8005924 33782057

[23] Akpınar M, Salmanpour F. Chat Generative Pretrained Transformer-4.0’s accuracy in assessing cervical vertebrae and hand-wrist maturation stages: A retrospective study. Am J Orthod Dentofac Orthop. 2025;168(6):753–63. Epub 2025 Sep 29. PMID: 41026070. 10.1016/j.ajodo.2025.08.010 41026070

[24] Gonca M, Sert MF, Gunacar DN, Kose TE, Beser B. Determination of growth and developmental stages in hand–wrist radiographs: can fractal analysis in combination with artificial intelligence be used? J Orofac Orthop. 2024;85(Suppl 2):1–15. 10.1007/s00056-023-00510-1. 38252312 10.1007/s00056-023-00510-1

[25] Jiang F, Abdulqader AA, Yan Y, Cheng F, Xiang T, Yu J, Li J, Qiu Y, Chen X. Deep learning based quantitative cervical vertebral maturation analysis. Head Face Med. 2025;21(1):20. 10.1186/s13005-025-00498-6 . PMID: 40140932; PMCID: PMC11938625. 40140932 10.1186/s13005-025-00498-6 PMC11938625

[26] Kanchanapiboon P, Tunksook P, Ritthipravat P, Boonpratham S, Satravaha Y, Chaweewannakorn C, Peanchitlertkajorn S. Classification of cervical vertebral maturation stages with machine learning models: leveraging datasets with high inter- and intra-observer agreement. Prog Orthod. 2024;25(1):35. 10.1186/s40510-024-00535-1. 39279025 10.1186/s40510-024-00535-1 PMC11402886

[27] Kavasoglu N, Ertugrul OF, Kotan S, Hazar Y, Eratilla V. Artificial Intelligence-Assisted Wrist Radiography Analysis in Orthodontics: Classification of Maturation Stage. Applied Sciences. 2025;15(21):11681. Available from: https://www.mdpi.com/2076-3417/15/21/11681.

[28] Khazaei M, Mollabashi V, Khotanlou H, Farhadian M. Automatic determination of pubertal growth spurts based on the cervical vertebral maturation staging using deep convolutional neural networks. J World Fed Orthod. 2023;12(2):56–63. 10.1016/j.ejwf.2023.02.003. 36890034 10.1016/j.ejwf.2023.02.003

[29] Kim EG, Oh IS, So JE, Kang J, Le VNT, Tak MK, Lee DW. Estimating cervical vertebral maturation with a lateral cephalogram using the convolutional neural network. J Clin Med. 2021;10(22):5400. 10.3390/jcm10225400. 34830682 10.3390/jcm10225400 PMC8620598

[30] Kim DW, Kim J, Kim T, Kim YJ, Song IS, Ahn B, Choo J, Lee DY. Prediction of hand–wrist maturation stages based on cervical vertebrae images using artificial intelligence. Orthod Craniofac Res. 2021;24(Suppl 2):68–75. 10.1111/ocr.12514. 34405944 10.1111/ocr.12514

[31] Kim H, Kim CS, Lee JM, Lee JJ, Lee J, Kim JS, Choi SH. Prediction of Fishman’s skeletal maturity indicators using artificial intelligence. Sci Rep. 2023;13(1):5870. 10.1038/s41598-023-33058-6. 37041244 10.1038/s41598-023-33058-6 PMC10090071

[32] Kök H, İzgi MS, Acılar AM. Evaluation of the artificial neural network and Naive Bayes models trained with vertebra ratios for growth and development determination. Turk J Orthod. 2020;34(1):2–9. PMID: 33828872; PMCID: PMC7990271. 33828872 10.5152/TurkJOrthod.2020.20059 PMC7990271

[33] Kök H, Izgi MS, Acilar AM. Determination of growth and development periods in orthodontics with artificial neural network. Orthod Craniofac Res. 2021;24(Suppl 2):76–83. 10.1111/ocr.12443. 33232582 10.1111/ocr.12443

[34] Li H, Li H, Yuan L, Liu C, Xiao S, Liu Z, Zhou G, Dong T, Ouyang N, Liu L, Ma C, Feng Y, Zheng Y, Xia L, Fang B. The psc-CVM assessment system: a three-stage type system for CVM assessment based on deep learning. BMC Oral Health. 2023;23(1):557. 10.1186/s12903-023-03266-7. 37573308 10.1186/s12903-023-03266-7 PMC10422791

[35] Liu X, Wang R, Jiang W, Lu Z, Chen N, Wang H. Automated distal radius and ulna skeletal maturity grading from hand radiographs with an attention multi-task learning method. Tomography (Ann Arbor). 2024;10(12):1915–29. 10.3390/tomography10120139. 10.3390/tomography10120139 PMC11679689 39728901

[36] Mohammed MH, Omer ZQ, Aziz BB, Abdulkareem JF, Mahmood TMA, Kareem FA, Mohammad DN. Convolutional Neural Network-Based Deep Learning Methods for Skeletal Growth Prediction in Dental Patients. J Imaging. 2024;10(11):278. 10.3390/jimaging10110278 . PMID: 39590742; PMCID: PMC11595330. 39590742 10.3390/jimaging10110278 PMC11595330

[37] Mohammad-Rahimi H, Motamadian SR, Nadimi M, Hassanzadeh-Samani S, Minabi MAS, Mahmoudinia E, Lee VY, Rohban MH. Deep learning for the classification of cervical maturation degree and pubertal growth spurts: a pilot study. Korean J Orthod. 2022;52(2):112–22. 10.4041/kjod.2022.52.2.112. 35321950 10.4041/kjod.2022.52.2.112 PMC8964471

[38] Nogueira-Reis F, Cascante-Sequeira D, Farias-Gomes A, de Macedo MMG, Watanabe RN, Santiago AG, Tabchoury CPM, Freitas DQ. Determination of the pubertal growth spurt by artificial intelligence analysis of cervical vertebrae maturation in lateral cephalometric radiographs. Oral Surg Oral Med Oral Pathol Oral Radiol. 2024;138(2):306–15. 10.1016/j.oooo.2024.02.017. 38553310 10.1016/j.oooo.2024.02.017

[39] Ramnarayan BK, Sindhu P, Patil P, Krishnamurthy A, Mahesh DR, Darshana S. Development and validation of an artificial intelligence algorithm for cervical vertebral maturation staging using lateral cephalograms. Med J Armed Forces India. 2025;81:672–9. 10.1016/j.mjafi.2025.08.012. 41268013 10.1016/j.mjafi.2025.08.012 PMC12629825

[40] Shoari SA, Sadrolashrafi SV, Sohrabi A, Afrouzian R, Ebrahimi P, Kouhsoltani M, Soltani MK. Estimating mandibular growth stage based on cervical vertebral maturation in lateral cephalometric radiographs using artificial intelligence. Prog Orthod. 2024;25(1):28. 10.1186/s40510-024-00527-1. 38910180 10.1186/s40510-024-00527-1 PMC11194253

[41] Tentaş S, Özden S. Deep Learning Based Evaluation of Skeletal Maturation: A Comparative Analysis of Five Hand-Wrist Methods. Orthod Craniofac Res. 2025;28(6):943–54. 10.1111/ocr.70008 . Epub 2025 Jul 24. PMID: 40704688; PMCID: PMC12603682. 40704688 10.1111/ocr.70008 PMC12603682

[42] Yıldırım A, Cicek O, Genç YS. Can AI-Based ChatGPT Models Accurately Analyze Hand-Wrist Radiographs? A Comparative Study. Diagnostics (Basel). 2025;15(12):1513. 10.3390/diagnostics15121513 . PMID: 40564836; PMCID: PMC12191842. 40564836 10.3390/diagnostics15121513 PMC12191842

[43] Zhang Y, Lu Z, Zhou J, Sun Y, Yi W, Wang J, Du T, Li D, Zhao X, Xu Y, Li C, Qi K. CDSNet: an automated method for assessing growth stages from various anatomical regions in lateral cephalograms based on deep learning. J World Fed Orthod. 2025;14(3):154–60. 10.1016/j.ejwf.2024.09.007. 39578153 10.1016/j.ejwf.2024.09.007

[44] Yu AC, Mohajer B, Eng J. External Validation of Deep Learning Algorithms for Radiologic Diagnosis: A Systematic Review. Radiol Artif Intell. 2022;4(3):e210064. 10.1148/ryai.210064. 35652114 10.1148/ryai.210064 PMC9152694

[45] Mello-Thoms C, Mello CAB. Clinical applications of artificial intelligence in radiology. Br J Radiol. 2023;96(1150):20221031. 10.1259/bjr.20221031. 37099398 10.1259/bjr.20221031 PMC10546456

[46] Saw SN, Yan YY, Ng KH. Current status and future directions of explainable artificial intelligence in medical imaging. Eur J Radiol. 2025;183:111884. 10.1016/j.ejrad.2024.111884 . Epub 2024 Dec 6. 39667118 10.1016/j.ejrad.2024.111884

