# Supplementary material for: Artificial intelligence for predicting the pubertal growth spurt using cephalometric and hand–wrist radiographs: a systematic review and meta-analysis
Source: BMC Oral Health. 2026 Jun 3;26:1116. doi: 10.1186/s12903-026-08627-6 (PMC13295224; doi:10.1186/s12903-026-08627-6)
Supplement: Supplementary file 1 — Supplementary Material 1. [file 12903_2026_8627_MOESM1_ESM.pdf]

NIHR | National Institute for Health and Care Research

PROSPERO
International prospective register of systematic reviews

Pubertal Growth Spurt Prediction by Artificial Intelligence: A Systematic Review

Jordana Soares Chaves, Matheus de Lima Ruffini, Juliana Avila Duarte, Clara Takako Moriguchi

To enable PROSPERO to focus on COVID-19 submissions, this registration record has undergone basic automated checks for eligibility and is published exactly as submitted. PROSPERO has never provided peer review, and usual checking by the PROSPERO team does not endorse content. Therefore, automatically published records should be treated as any other PROSPERO registration. Further detail is provided here.

Citation

Jordana Soares Chaves, Matheus de Lima Ruffini, Juliana Avila Duarte, Clara Takako Moriguchi. Pubertal Growth Spurt Prediction by Artificial Intelligence: A Systematic Review. PROSPERO 2024 CRD42024594040. Available from https://www.crd.york.ac.uk/PROSPERO/view/CRD42024594040.

REVIEW TITLE AND BASIC DETAILS

Review title

Pubertal Growth Spurt Prediction by Artificial Intelligence: A Systematic Review

Original language title

Predição do Surto de Crescimento Puberal pela Inteligência Artificial: Uma Revisão Sistemática

Review objectives

What is the effectiveness of current artificial intelligence tools that use carpal and/or cephalometric radiographs in estimating the pubertal growth spurt?

Keywords

artificial intelligence, cephalometry, diagnostic imaging, hand and wrist, machine learning, Pubertal growth spurt

SEARCHING AND SCREENING

Searches

PubMed, Embase, Web of Science, LILACS

Study design

Both randomized and nonrandomized study types will be included.

Link to search strategy

A full search strategy has been uploaded to PROSPERO. The PDF may be accessed through this link https://www.crd.york.ac.uk/PROSPEROFILES/ec3e9cd0f6e88d41f3b8887c35aad71b.pdf.

ELIGIBILITY CRITERIA

Condition or domain being studied

The pubertal growth spurt represents a critical landmark in the assessment of skeletal maturation, exhibiting significant relevance in the fields of orthodontics, facial orthopedics, and pediatrics. Accurate prediction of this event is instrumental in determining the optimal timing for clinical intervention. Conventionally, monitoring relies on radiographic analyses interpreted manually, such as the assessment of cervical vertebral maturation stages and skeletal maturity stages of the hand and wrist.

The advent of digital health technologies has led to the application of artificial intelligence models to automate and refine this prediction process, potentially offering improved accuracy and reduced inter-observer variability. However, the extant scientific literature remains fragmented, lacking a consolidated analysis comparing the efficacy of these AI models.

Consequently, this systematic review and meta-analysis aims to identify and evaluate the performance of AI models employed to predict the pubertal growth spurt, utilizing data from cephalometric and hand-wrist radiographs

Population

Included

- Studies evaluating individuals up to 21 years of age.

Excluded

- Populations with specific clinical conditions, such as growth disorders, endocrine diseases, genetic syndromes, fractures, or other pathological bone alterations

Intervention(s) or exposure(s)

Included

- Studies that utilize artificial intelligence models, such as Machine Learning, Deep Learning, or Convolutional Neural Networks;
- Studies that use cephalometric radiographs (lateral cephalograms) or hand-wrist radiographs (carpal);
- Artificial intelligence models focused on:
    - Direct prediction of the pubertal growth spurt, or
    - Prediction of the peak of the pubertal growth spurt, or

    - Classification of validated skeletal maturation stages, provided there is an association with the period of the growth spurt or peak;
- Studies providing details on the methodology used and algorithms employed.

Excluded

- Artificial intelligencemodels applied exclusively to the prediction of chronological bone age, without direct association to the pubertal growth spurt or peak;
- Studies that do not utilize artificial intelligence;
- Studies limited to anatomical segmentation or image processing tasks, without performing functional prediction;
- Studies that use other imaging modalities, such as magnetic resonance imaging or computed tomography.

Comparator(s) or control(s)

Included
When available, the comparator will be the traditional manual methods for assessing the pubertal growth spurt or skeletal maturity, including cervical vertebral maturation (CVM) and hand-wrist radiograph-based methods. Studies without a direct comparator will still be included.

Context

This review will include studies involving human participants aged up to 21 years. Eligible studies must apply artificial intelligence (AI) models—such as machine learning, deep learning, or convolutional neural networks—for the analysis of radiographic images. Only studies that use lateral cephalometric radiographs and/or hand-wrist radiographs as input data will be considered.

The included AI models should aim to: (i) directly predict the pubertal growth spurt (PGS), (ii) predict the peak of the pubertal growth spurt, or (iii) classify validated skeletal maturation stages that are explicitly associated with the pubertal growth period.

Furthermore, studies must provide sufficient methodological detail, including the performance metrics reported (e.g., accuracy, sensitivity, specificity, area under the curve). Only full-text articles written in English, Portuguese, or Spanish will be included.

OUTCOMES TO BE ANALYSED

Main outcomes

The outcomes are the performance metrics of AI models developed to predict the pubertal growth spurt or classify skeletal maturation stages related to it. These include sensitivity, specificity, area under the ROC curve (AUC), accuracy, F1-score, mean absolute error (MAE) and root mean square error (RMSE). Information about AI models will also be extracted, including the type of algorithm, input radiographs (lateral cephalometric and/or hand-wrist), and whether the model predicts the pubertal growth spurt directly or indirectly. The effect measures for synthesis will include pooled sensitivity, specificity, AUC, MAE, RMSE, and correlation coefficients when data are sufficiently homogeneous.

Additional outcomes

No additional results

DATA COLLECTION PROCESS

Data extraction (selection and coding)

The selection of studies will follow a systematic two-stage screening procedure. Initially, screening will focus on the titles and abstracts of the publications found in the initial search. Relevant studies will be retained for further evaluation. In the next stage, a detailed analysis of the full texts of the selected studies will be carried out. Inclusion in this phase will depend on meeting specific criteria, the availability of the necessary information on the results, as well as access to the full texts.The selection of studies will be carried out independently by at least two reviewers, who will compare the information collected. Discrepancies in the selection process will be resolved by consensus or after consulting a third reviewer when necessary.The data that will be collected will include essential information such as the title, the authors, the year of publication, the country of origin, the type of study and the methodology used. Specific data will also be recorded in relation to the main results and other relevant metrics, such as measurements of the model's effectiveness, details of the AI algorithms implemented, radiographs selected, image pre-processing techniques used, validation method, as well as ethical and regulatory compliance considerations.The extracted data will be added to a standardized form, where it will be systematically recorded. After collection, the extracted data will be organized in a spreadsheet or database to facilitate subsequent analysis.

Risk of bias (quality) assessment

The following study characteristics will be assessed during data extraction and synthesis: study design, population characteristics (e.g., age, sex, sample size), type and source of radiographic images (cephalometric or hand-wrist), AI model type (e.g., machine learning, deep learning, convolutional neural networks), reference standard used for SCP identification (e.g., CVM or HWM stages), predicted outcome (e.g., classification of growth stage, direct prediction of pubertal peak), and performance metrics (e.g., accuracy, sensitivity, specificity, AUC).

The risk of bias and applicability of the included studies will be assessed using the QUADAS-AI tool, which is an extension of the QUADAS-2 instrument, specifically adapted for evaluating diagnostic accuracy studies involving artificial intelligence. Two reviewers will independently perform the assessment, with disagreements resolved by discussion or third-party adjudication.

PLANNED DATA SYNTHESIS

Strategy for data synthesis

A narrative synthesis will be conducted to summarize and compare the characteristics of the included studies, including AI model types, input data (radiographic modality), reference standards, outcome definitions, and study settings.

When at least two studies report comparable performance metrics for similar prediction targets, a meta-analysis will be performed. The primary outcome will be the predictive accuracy of AI models for the timing or presence of the pubertal growth spurt.

For quantitative synthesis, a bivariate random-effects model will be applied when sensitivity and specificity are reported, enabling calculation of pooled estimates with 95% confidence intervals.

When only accuracy or AUC is reported, a random-effects meta-analysis of proportions or means will be used, depending on the metric.

Heterogeneity will be assessed using the I^2 statistic and visual inspection of forest plots. Subgroup analyses or meta-regression may be conducted based on input modality (cephalometric vs. hand-wrist), or type of AI model (e.g., deep learning vs. classical machine learning).

All analyses will be conducted using R (packages: meta, mada, or metafor) and/or RevMan, as appropriate for the data structure.

Analysis of subgroups or subsets

Subgroup analyses will be conducted to explore whether specific factors influence the predictive performance of AI models for pubertal growth spurt prediction. One planned analysis will compare studies using hand-wrist radiographs versus those using lateral cephalometric radiographs, to investigate whether the imaging modality impacts model performance. This analysis will be conducted only if a sufficient number of studies are available for each modality.

A second subgroup analysis will compare the type of deep learning algorithm used, such as Convolutional Neural Networks (CNNs) versus Recurrent Neural Networks (RNNs), where applicable.

For each subgroup, performance metrics (e.g., accuracy, sensitivity, specificity, AUC) will be synthesized. Stratified meta-analyses or meta-regression will be used to assess statistical significance when appropriate.

REVIEW AFFILIATION, FUNDING AND PEER REVIEW

Review team members

Miss Jordana Soares Chaves (review guarantor and contact) Hospital de Clínicas de Porto Alegre. Brazil.

No conflict of interest declared.

Matheus de Lima Ruffini. UFRGS. Brazil.

No conflict of interest declared.

Dr Juliana Avila Duarte. Hospital de Clínicas de Porto Alegre. Brazil.

No conflict of interest declared.

Clara Takako Moriguchi. UFRGS. Brazil.

No conflict of interest declared.

Named contact

Miss Jordana Soares Chaves (jschaves@hcpa.edu.br). Hospital de Clínicas de Porto Alegre. Brazil.

Review affiliation

Hospital de Clínicas de Porto Alegre (HCPA)

Universidade Federal do Rio Grande do Sul (UFRGS)

Funding source
the study is not funded

TIMELINE OF THE REVIEW

Review timeline
Start date: 01 October 2024. End date: 01 October 2025

Date of first submission to PROSPERO
20 October 2024

Date of registration in PROSPERO
31 October 2024

CURRENT REVIEW STAGE

Publication of review results
The intention is to publish the review once completed.The review will be published in English

Stage of the review at this submission 1 change

Review stage	Started	Completed
Pilot work	✓	✓
Formal searching/study identification	✓	✓
Screening search results against inclusion criteria	✓	✓
Data extraction or receipt of IPD	✓	✓
Risk of bias/quality assessment	✓	✓
Data synthesis	✓	✓

Review status
The review is completed.

ADDITIONAL INFORMATION

PROSPERO version history 1 change

- Version 3.1, published 29 Oct 2025
- Version 3.0, published 12 Sep 2025
- Version 2.0, published 12 Sep 2025
- Version 1.1, published 31 Oct 2024
- Version 1.0, published 31 Oct 2024

Review conflict of interest
None known

Country
Brazil

Medical Subject Headings
Diagnosis, Computer-Assisted; Artificial Intelligence; Deep Learning; Neural Networks, Computer; Radiography; Puberty; Cervical Vertebrae; Hand; Wrist; Growth and Development; Orthodontics

Revision note 1 change
The review has been completed

Disclaimer
The content of this record displays the information provided by the review team. PROSPERO does not peer review registration records or endorse their content.

PROSPERO accepts and posts the information provided in good faith; responsibility for record content rests with the review team. The guarantor for this record has affirmed that the information provided is truthful and that they understand that deliberate provision of inaccurate information may be construed as scientific misconduct.

PROSPERO does not accept any liability for the content provided in this record or for its use. Readers use the information provided in this record at their own risk.

Any enquiries about the record should be referred to the named review contact
