# Supplementary material for: Artificial intelligence for predicting the pubertal growth spurt using cephalometric and hand–wrist radiographs: a systematic review and meta-analysis
Source: BMC Oral Health. 2026 Jun 3;26:1116. doi: 10.1186/s12903-026-08627-6 (PMC13295224; doi:10.1186/s12903-026-08627-6)

**SUPPLEMENTARY MATERIALS**

**Supplementary Table S1. Full electronic search strategies for all databases**

| **Database** | **Search** |
| --- | --- |
| PUBMED | (Puberty[mh:noexp] OR Puber*[tiab] OR Age Determination by Skeleton[mh] OR Age Determination[tiab] OR Skeletal Age[tiab] OR Bone Age[tiab] OR Skeletal Growth[tiab] OR Bone growth[tiab] OR Skeletal Maturation[tiab] OR Bone Maturation[tiab])  AND  (Wrist/diagnostic imaging[mh] OR Hand/diagnostic imaging[mh] OR Skull/diagnostic imaging[mh]  OR Cephalometry[mh] OR Teleradiology[mh] OR Cephalometr*[tiab] OR Craniometr*[tiab] OR  Lateral teleradiograph*[tiab] OR ((Wrist*[tiab] OR Hand[tiab] OR Hands[tiab] OR Skull*[tiab])  AND (Radiograph*[tiab] OR Radiolog*[tiab] OR X ray[tiab] OR X rays[tiab] OR teleradiograph*[tiab] OR teleradiolog*[tiab] OR imag*[tiab])))  AND  (Artificial Intelligence[mh] OR Artificial Intelligence*[tiab] OR AI[tiab] OR Machine Learning[tiab]  OR Deep learning[tiab] OR Neural network*[tiab] OR Convolutional network*[tiab]) |
| EMBASE | (Puberty/de OR Puber*:ti,ab,kw OR 'bone age determination'/exp OR 'Age Determination':ti,ab,kw OR 'Skeletal Age':ti,ab,kw OR 'Bone Age':ti,ab,kw OR 'Skeletal Growth':ti,ab,kw OR  'Bone growth':ti,ab,kw OR 'Skeletal Maturation':ti,ab,kw OR 'Bone Maturation':ti,ab,kw)  AND  (((wrist/exp OR Hand/exp OR skull/exp) AND 'dignostic imaging'/exp) OR Cephalometry/exp OR  Teleradiology/exp OR Cephalometr*:ti,ab,kw OR Craniometr*:ti,ab,kw OR 'Lateral teleradiograph*':ti,ab,kw OR ((Wrist*:ti,ab,kw OR Hand:ti,ab,kw OR Hands:ti,ab,kw OR Skull*:ti,ab,kw)  AND (Radiograph*:ti,ab,kw OR Radiolog*:ti,ab,kw OR X ray:ti,ab,kw OR X rays:ti,ab,kw OR teleradiograph*:ti,ab,kw OR teleradiolog*:ti,ab,kw OR imag*:ti,ab,kw)))  AND  ('Artificial Intelligence'/exp OR 'Artificial Intelligence*':ti,ab,kw OR AI:ti,ab,kw OR 'Machine Learning':ti,ab,kw OR 'Deep learning':ti,ab,kw OR 'Neural network*':ti,ab,kw OR 'Convolutional  network*':ti,ab,kw)  AND  [embase]/lim NOT ([embase]/lim AND [medline]/lim) |
| WEB OF SCIENCE | (TI=(Puber* OR "Age Determination by Skeleton" OR "Age Determination" OR "Skeletal Age" OR "Bone Age" OR "Skeletal Growth" OR "Bone growth" OR "Skeletal Maturation" OR "Bone Maturation") OR AB=(Puber* OR "Age Determination by Skeleton" OR "Age Determination" OR "Skeletal Age" OR "Bone Age" OR "Skeletal Growth" OR "Bone growth" OR "Skeletal Maturation" OR "Bone Maturation") OR AK=(Puber* OR "Age Determination by Skeleton" OR "Age Determination" OR "Skeletal Age" OR "Bone Age" OR "Skeletal Growth" OR "Bone growth" OR "Skeletal Maturation" OR "Bone Maturation")) AND (TI=(((wrist OR Hand OR skull) AND "dignostic imaging") OR Cephalometr* OR Teleradiology OR Craniometr* OR "Lateral teleradiograph*" OR ((Wrist* OR Hand OR Hands OR Skull*) AND (Radiograph* OR Radiolog* OR "X ray" OR "X rays" OR teleradiograph* OR teleradiolog* OR imag*))) OR AB=(((wrist OR Hand OR skull) AND "dignostic imaging") OR Cephalometr* OR Teleradiology OR Craniometr* OR "Lateral teleradiograph*" OR ((Wrist* OR Hand OR Hands OR Skull*) AND (Radiograph* OR Radiolog* OR "X ray" OR "X rays" OR teleradiograph* OR teleradiolog* OR imag*))) OR AK=(((wrist OR Hand OR skull) AND "dignostic imaging") OR Cephalometr* OR Teleradiology OR Craniometr* OR "Lateral teleradiograph*" OR ((Wrist* OR Hand OR Hands OR Skull*) AND (Radiograph* OR Radiolog* OR "X ray" OR "X rays" OR teleradiograph* OR teleradiolog* OR imag*)))) AND (TI=("Artificial Intelligence" OR AI OR "Machine Learning" OR "Deep learning" OR "Neural network*" OR "Convolutional network*") OR AB=("Artificial Intelligence" OR AI OR "Machine Le- arning" OR "Deep learning" OR "Neural network*" OR "Convolutional network*") OR AK=("Artificial Intelligence" OR AI OR "Machine Learning" OR "Deep learning" OR "Neural network*" OR "Convolutional network*")) |
| LILACS | (mh:G08.686.760 OR E01.370.049* OR ti:(Puber* OR "Determinação da Idade pelo Esqueleto" OR "Medida da idade pelo esqueleto" OR "Medida da idade pelos ossos" OR "Determinación de la Edad por el Esqueleto" OR "Medida de la Edad por el Esqueleto" OR "Medida de la Edad por los Huesos" OR "Age Determination by Skeleton" OR "Age Determination" OR "Skeletal Age" OR "Bone Age" OR "Skeletal Growth" OR "Bone growth" OR "Skeletal Maturation" OR "Bone Maturation") OR ab:(Puber* OR "Determinação da Idade pelo Esqueleto" OR "Medida da idade pelo esqueleto" OR "Medida da idade pelos ossos" OR "Determinación de la Edad por el Esqueleto" OR "Medida de la Edad por el Esqueleto" OR "Medida de la Edad por los Huesos" OR "Age Determination by Skeleton" OR "Age Determination" OR "Skeletal Age" OR "Bone Age" OR "Skeletal Growth" OR "Bone growth" OR "Skeletal Maturation" OR "Bone Maturation")) AND (mh:(Wrist/DG OR Hand/DG OR Skull/DG OR Cephalometry OR Teleradiology) OR ti:(Cefalometria OR "Circunferência Craniana" OR Craniometria OR "Perímetro Cefálico" OR craneometria OR Cephalometr* OR Craniometr* OR Lateral teleradiograph* OR ((punho OR muñeca OR Wrist* OR mãos OR mano OR Hand OR Hands OR Crani* OR "calota craniana" OR calvaria OR craneo OR "calota craneal" OR Skull*) AND (radiografia OR Roentgenografia OR Radiograph* OR Radiolog* OR "X ray" OR "X rays" OR teleradiograph* OR teleradiolog* OR imag*))) OR ab:(Cefalometria OR "Circunferência Craniana" OR Craniometria OR "Perímetro Cefálico" OR craneometria OR Cephalometr* OR Craniometr* OR Lateral teleradiograph* OR ((punho OR muñeca OR Wrist* OR mãos OR mano OR Hand OR Hands OR Crani* OR "calota craniana" OR calvaria OR craneo OR "calota craneal" OR Skull*) AND (radiografia OR Roentgenografia OR Radiograph* OR Radiolog* OR "X ray" OR "X rays" OR teleradiograph* OR teleradiolog* OR imag*)))) AND (mh:G17.035.250 OR ti:("Inteligência artificial" OR "Artificial Intelligence*" OR AI OR "Machine Learning" OR "Deep learning" OR "Neural network*" OR "Convolutional network*") OR ab:("Inteligência artificial" OR "Artificial Intelligence*" OR AI OR "Machine Learning" OR "Deep learning" OR "Neural network*" OR "Convolutional network*")) AND db:("LILACS") |

**Supplementary Figure S1 – HSROC curve for cervical vertebral maturation (CVM)-based studies**


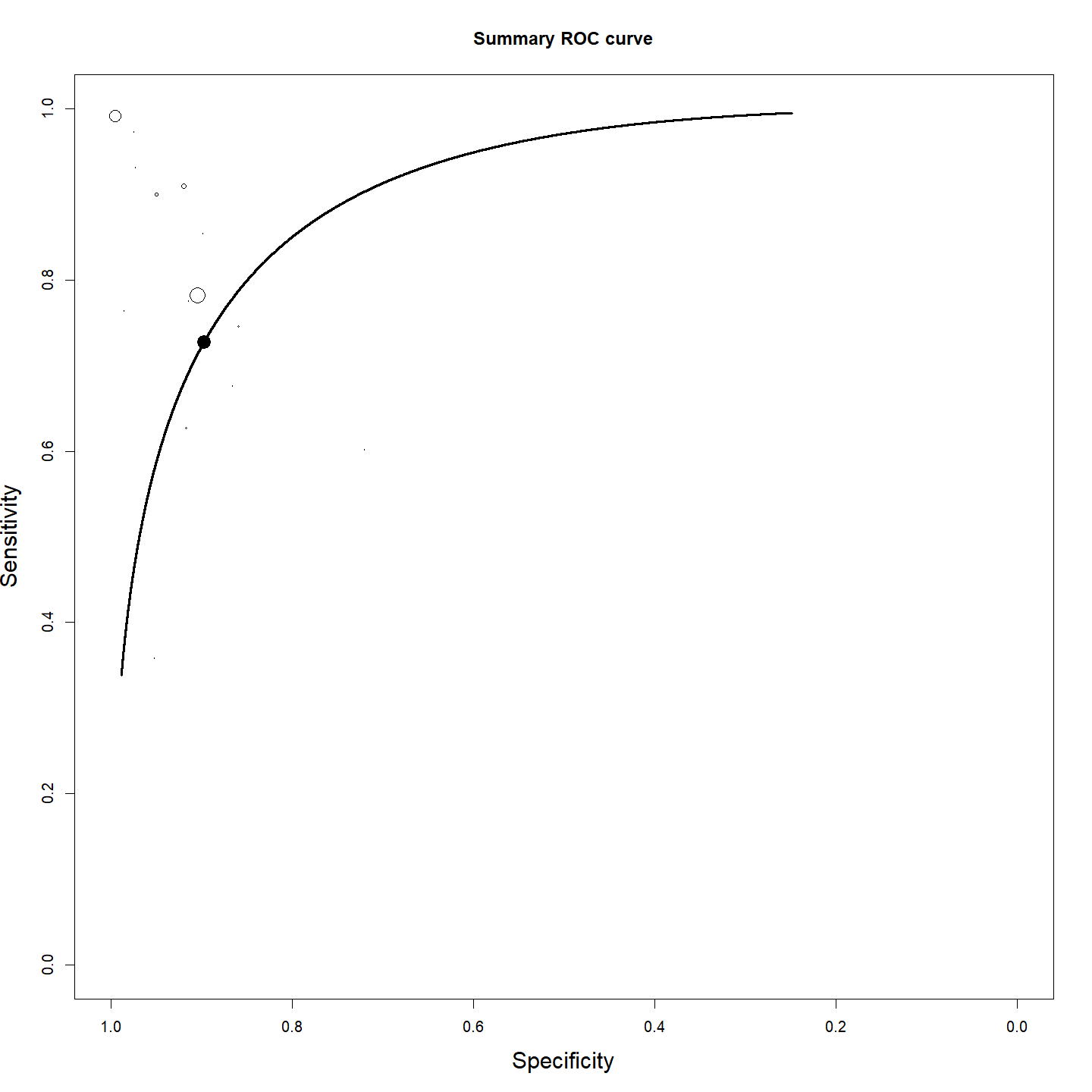


**Supplementary Figure S2 – HSROC curve for hand–wrist-based studies**
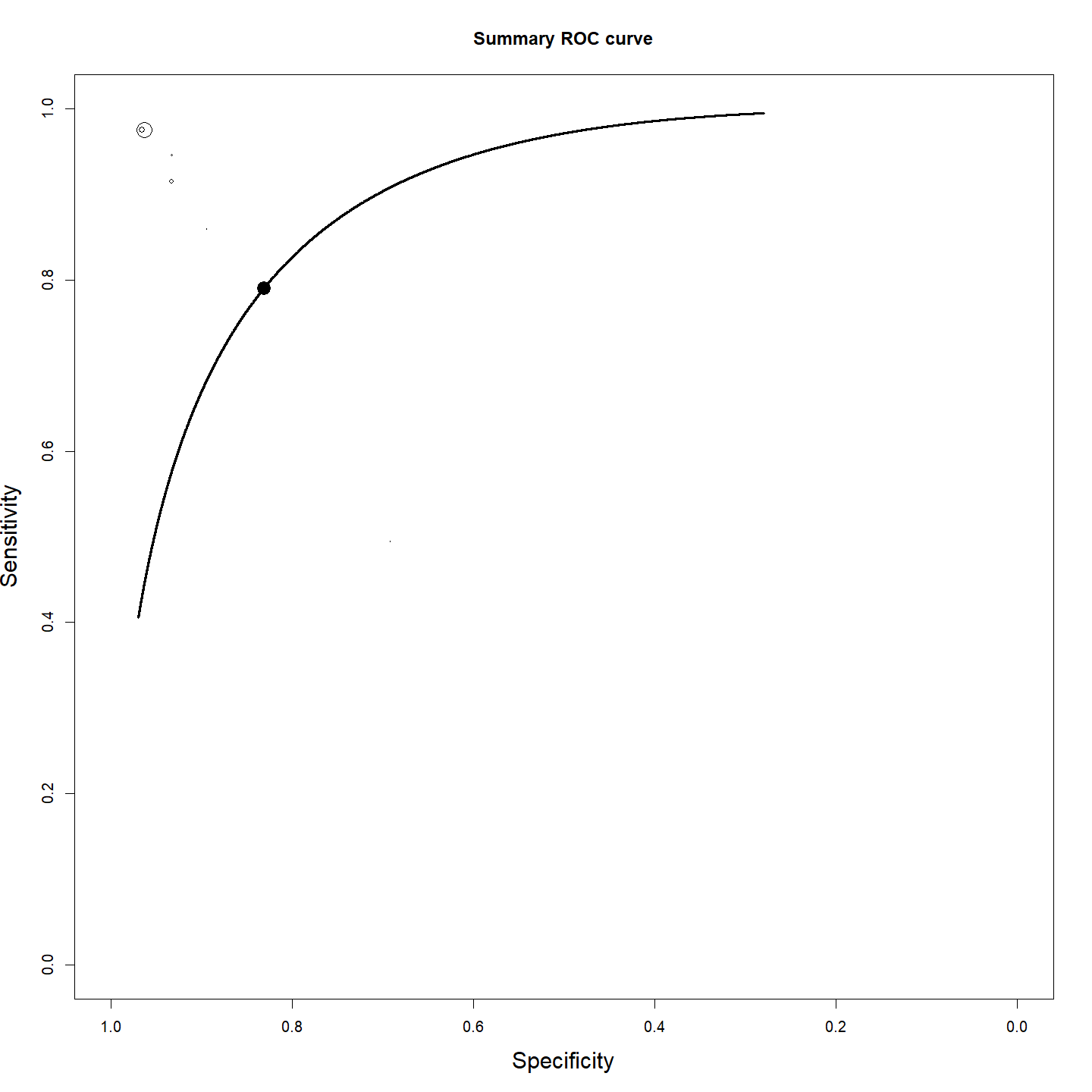


**Supplementary Figure S3 – HSROC curve for deep learning models (CNN, Transformer, and YOLO)**
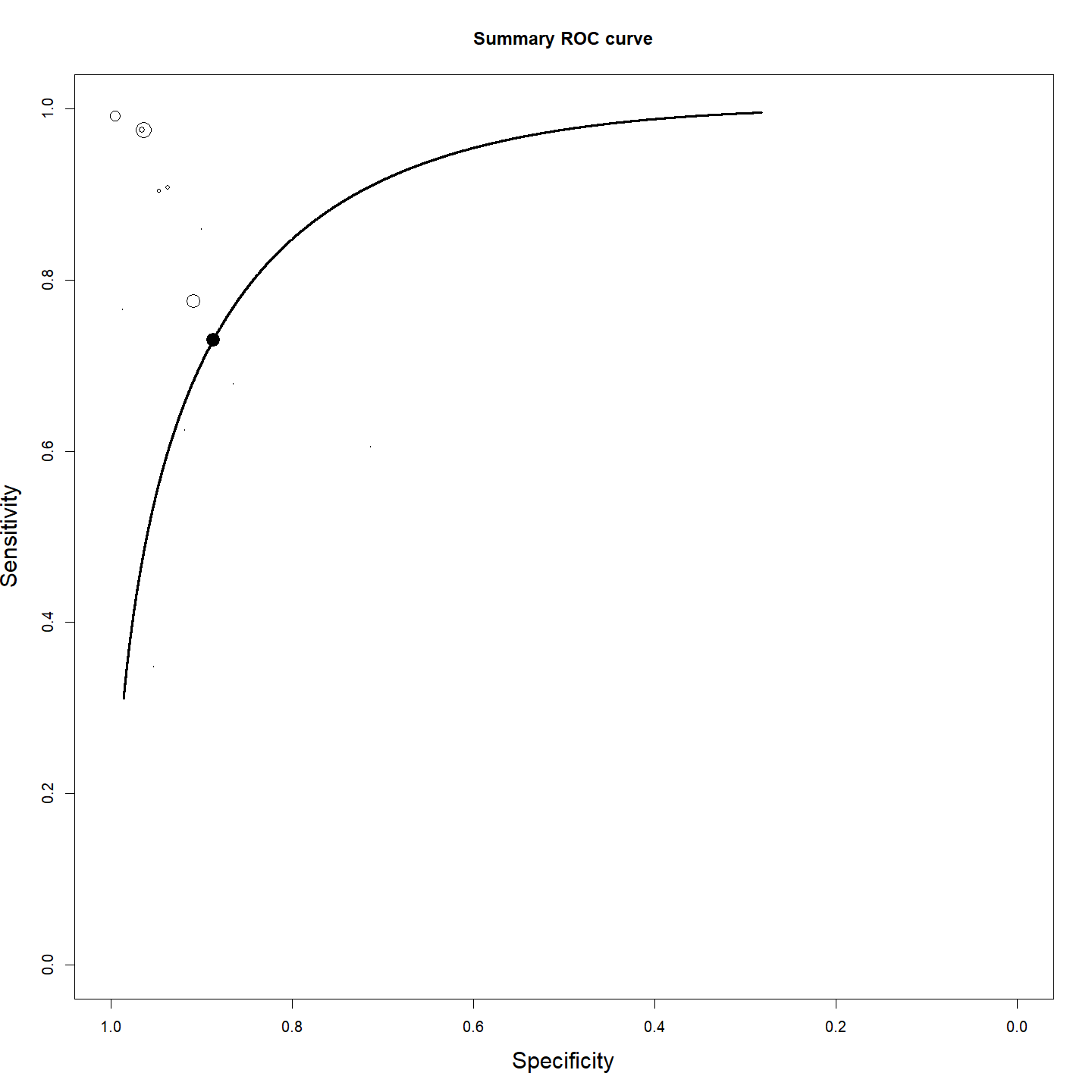


**Supplementary Figure S4 – HSROC curve for traditional machine learning models (SVM, MLP, and ensemble methods)**


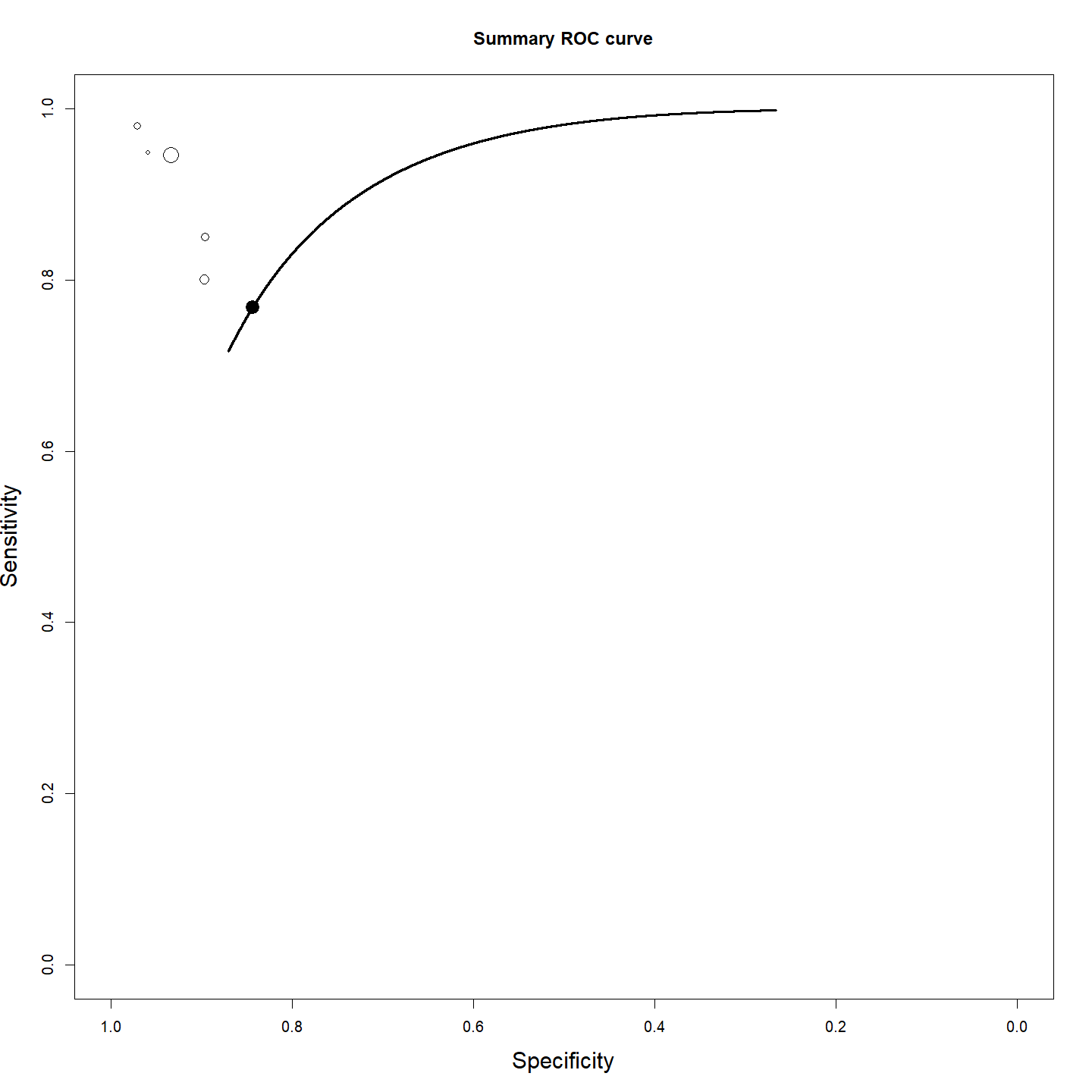


**Supplementary Figure S5 – HSROC curve for large language model (LLM)-based approaches**
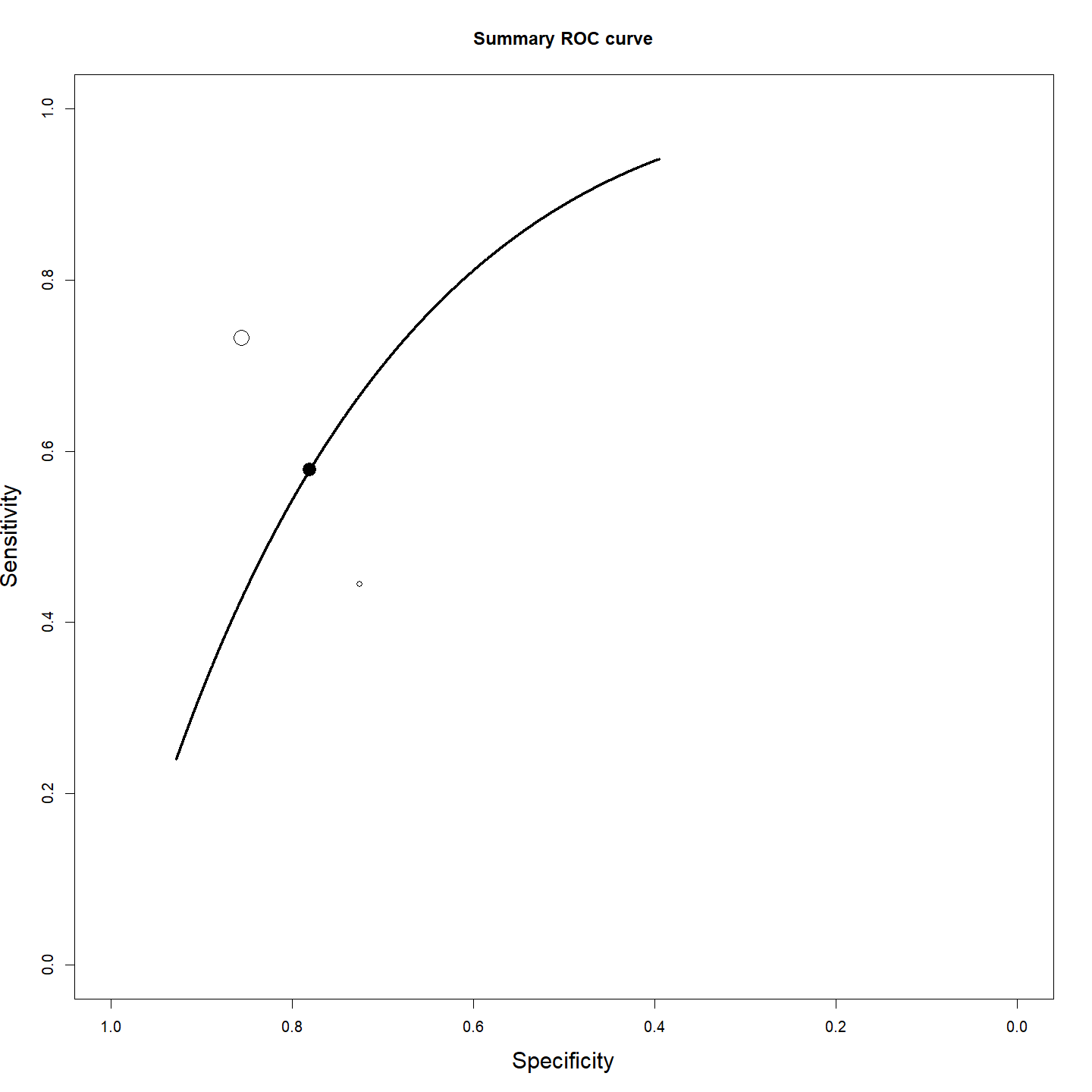


**Supplementary Figure S6 – Bar Plot of QUADAS-AI Summary Risk-of-Bias Ratings**
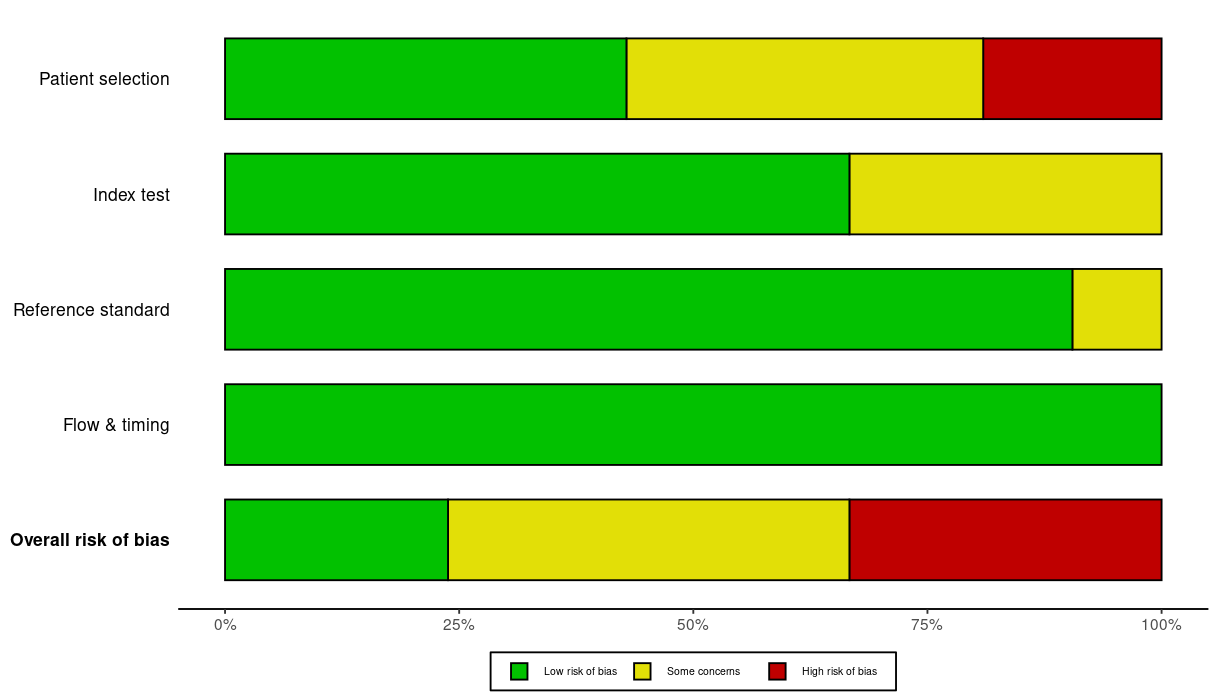

Supplement: Supplementary file 3 — Supplementary Material 3: Supplementary Table S1. Full electronic search strategies for all databases. Supplementary Figure S1 – HSROC curve for cervical vertebral maturation (CVM)-based studies. Supplementary Figure S2 – HSROC curve for hand–wrist-based studies. Supplementary Figure S3 – HSROC curve for deep learning models (CNN, Transformer, and YOLO). Supplementary Figure S4 – HSROC curve for traditional machine learning models (SVM, MLP, and ensemble methods). Supplementary Figure S5 – HSROC curve for large language model (LLM)-based approaches. Supplementary Figure S6 – Bar Plot of QUADAS-AI Summary Risk-of-Bias Ratings. [file 12903_2026_8627_MOESM3_ESM.docx]
